# Deep Biological Clocks in Critical Care Medicine: A Scoping Review Toward Translational Precision Care

**DOI:** 10.3390/jpm16020092

**Published:** 2026-02-04

**Authors:** Ithamar Cheyne, Magdalena Voinič, Tara Radaideh, Abdullah Daher, Julia Niezgoda, Maja Anna Romanowska, Małgorzata Mikaszewska-Sokolewicz

**Affiliations:** 1 Anesthesiology and Critical Care Scientific Circle English Division (ANKONA ED), Medical University of Warsaw, 02-004 Warsaw, Poland; 2 Department of Anesthesiology and Intensive Care, Children’s Memorial Health Institute, 04-730 Warsaw, Poland

**Keywords:** intensive care units, aging, DNA methylation, telomere length, cell-free DNA, epigenomics, critical illness, sepsis, prognosis, precision medicine

## Abstract

**Background**: Outcomes after critical illness vary markedly despite similar diagnoses and severity scores, underscoring the limitations of chronological age and conventional Intensive Care Unit (ICU) prognostic tools. Personalization of critical care is increasingly essential to improve not only short-term survival but also long-term post-discharge outcomes. Biological aging clocks provide a quantitative framework to capture physiological reserve, immune competence, and vulnerability to stress. **Methods**: We conducted a scoping review of original human studies published between January 2015 and October 2025 that evaluated biological aging biomarkers in adult ICU populations. PubMed/MEDLINE, Scopus, Web of Science, and Embase were searched, with backward citation screening. **Results**: Across epigenetic, telomere-based, cfDNA, proteomic, metabolomic, and phenotypic aging measures, accelerated biological aging was consistently associated with increased mortality, organ dysfunction, and post-ICU vulnerability. Despite substantial methodological heterogeneity, a convergent signal emerged linking inflammation-weighted and stress-responsive deep biological clocks to clinically meaningful outcomes in critically ill patients. **Conclusions**: Biological aging biomarkers represent a mechanistically grounded approach to personalized prognostication in critical care. From a translational perspective, deep biological clocks hold promise for personalized risk stratification, prognostication, and the identification of high-risk recovery phenotypes, although prospective validation and implementation studies are required.

## 1. Introduction

Advances in critical care medicine have substantially improved short-term survival from acute life-threatening conditions; however, morbidity, long-term disability, and post-ICU vulnerability remain disproportionately high, particularly among older and frail patients [1]. Chronological age alone has proven insufficient to explain the marked heterogeneity in outcomes observed among critically ill individuals with comparable disease severity [2]. This has prompted growing interest in biological aging as a framework for better capturing interindividual differences in physiological reserve, stress response, and recovery capacity.

Biological age refers to the cumulative functional state of multiple organ systems and can be quantified using molecular and phenotypic biomarkers that diverge from chronological age. Throughout this review, the term ‘deep biological clocks’ refers to integrative, multi-domain aging estimators, often multi-omic or composite, that aim to capture systemic biological stress and aging-related vulnerability. Among the most extensively studied approaches are epigenetic clocks derived from DNA methylation patterns, including the original Horvath clock [3] and second-generation clocks such as PhenoAge [4] which incorporate inflammation- and metabolism-related biomarkers and show stronger associations with morbidity and mortality [5]. These measures have been validated across large population cohorts and are consistently associated with all-cause mortality, cardiovascular disease, and functional decline. Parallel aging biomarkers—including telomere length (TL) dynamics, circulating cell-free DNA (cfDNA), and multi-omic, proteomic and metabolomic signatures—capture complementary dimensions of cellular turnover, senescence, and tissue injury [6]

Critical illness represents a unique biological context in which baseline aging trajectories intersect with profound acute systemic stress. In particular, sepsis, acute respiratory distress syndrome (ARDS), trauma, cardiogenic shock, and major surgery induce widespread inflammation, endothelial dysfunction, mitochondrial stress, and metabolic reprogramming—processes that overlap mechanistically with established hallmarks of aging [7]. Experimental and clinical data suggest that acute inflammatory stress can transiently or persistently accelerate molecular aging signatures, particularly those reflected by epigenetic clocks and proteomic stress networks, thereby amplifying vulnerability beyond that predicted by pre-illness health status alone [5]. At the same time, biological aging markers appear sensitive to recovery and resolution of systemic stress, indicating that aging acceleration in the ICU may be dynamic rather than strictly unidirectional.

Despite these advances, the role of biological clocks in critical care remains fragmented across disease-specific studies and biomarker modalities. Existing ICU prognostic tools—such as Acute Physiology and Chronic Health Evaluation II (APACHE II) and Sequential Organ Failure Assessment (SOFA)—are optimized to quantify acute physiological derangement but do not incorporate molecular indicators of resilience, senescence, or long-term vulnerability. As a result, they incompletely explain why patients with similar severity scores experience markedly different outcomes, trajectories of organ failure, or post-ICU recovery [8]. Integrating biological aging metrics into critical care research, therefore, offers an opportunity to bridge chronic health status with acute pathophysiology, potentially improving risk stratification and mechanistic understanding.

To address this gap, we conceptualize biological aging in the ICU as the convergence of two forces: baseline biological vulnerability (shaped by frailty, comorbidity burden, body composition, and immunosenescence) and acute systemic stress induced by critical illness. This interaction drives dynamic biological age acceleration detectable across multiple complementary modalities, including epigenetic clocks, cfDNA-based tissue injury signals, and proteomic–metabolomic stress networks. These integrated signals, in turn, are associated with clinically meaningful outcomes such as mortality, organ dysfunction, length of stay, and impaired recovery.

The objective of this scoping review is to synthesize current evidence on biological clocks in the ICU, focusing on (i) the consistency of associations between biological aging metrics and critical care outcomes, (ii) disease-specific versus shared aging signatures across systemic ICU pathologies, and (iii) methodological limitations and translational challenges that currently restrict clinical implementation. By framing biological aging as a dynamic systems-level process rather than a static attribute, this review aims to clarify how biological clocks may enhance personalized prognostic assessment and deepen mechanistic insight into critical illness.

Most prior reviews of biological aging clocks have focused on community or chronic disease populations and on clock development or long-term epidemiological associations. In contrast, this review focuses specifically on critically ill adults, a setting characterized by extreme acute stress and fundamentally different biological dynamics.

A recent narrative review [9] has discussed the concept of biological age in critical care, focusing on definitions, estimation methods, and potential clinical implications. However, this work does not systematically map the available evidence nor integrate the rapidly expanding literature across multiple biomarker domains. In contrast, the present scoping review applies a structured framework to comprehensively survey epigenetic, telomere-based, cell-free DNA, proteomic, metabolomic, transcriptomic, and phenotypic aging measures specifically in critically ill adults. By emphasizing ICU-specific populations and acute stress–induced aging dynamics within a translational precision-care perspective, this review provides a structured, multi-domain synthesis of current evidence and identifies key gaps for future clinical implementation.

## 2. Materials and Methods

This scoping review was conducted in accordance with the Preferred Reporting Items for Systematic Reviews and Meta-Analyses extension for Scoping Reviews (PRISMA-ScR) (Supplementary Material S1) [10]. The review followed a predefined methodological framework including systematic searching, study selection based on eligibility criteria, data charting, and narrative synthesis. No protocol was registered.

A comprehensive search strategy was developed to identify studies evaluating deep-aging biomarkers or biological age-estimation methods in critically ill adult populations. The complete keyword strategy and search code for each database, including all Boolean operators and individual search strings, are provided in Appendix A to ensure reproducibility and methodological transparency.

Searches were carried out across major biomedical databases, including PubMed/MEDLINE, Scopus, Web of Science, and Embase. Eligible studies had to: involve adults (≥18 years) with critical illness receiving care in an intensive care unit or equivalent acute-care setting; measure at least one aging biomarker or deep aging clock (such as DNA methylation markers, epigenetic clocks like PhenoAge, TL, cfDNA–derived aging indices, proteomic or metabolomic age signatures, or multi-omic biological age models); report one or more clinically relevant outcomes; and be original research, encompassing both prospective and retrospective study designs. Only studies published in English were included. The search was limited to articles published between 1 January 2015 and 31 October 2025, to ensure contemporary relevance and capture recent developments in deep-aging biomarker techniques. Studies were excluded if they were non-original publications (such as reviews, systematic reviews, meta-analyses, or editorials), not published in English or outside the specified date range, involved animals or pediatric populations, did not focus on critically ill adults, or did not measure an aging biomarker or deep aging clock.

All titles and abstracts identified through the search were screened for relevance, followed by full-text assessment of potentially eligible articles. Two reviewers independently screened, extracted, and categorized all eligible studies into disease- and population-specific sections and by aging clock domain, with disagreements resolved by consensus. Reasons for exclusion at the full-text stage were documented. The final set of included studies was determined after consensus review.

The results were synthesized narratively, with emphasis on mapping the breadth of aging biomarkers studied in critical illness, identifying shared patterns across similar pathologies, and highlighting methodological limitations and knowledge gaps within each biomarker category. Because this was a scoping review, no risk-of-bias assessment or evidence grading was performed, consistent with PRISMA-ScR recommendations. Before interpreting these findings, it is important to emphasize that the current evidence base is partially derived from small, single-center, observational studies with heterogeneous designs, limited longitudinal sampling, and variable adjustment for confounders. No quantitative synthesis or meta-analysis was planned due to expected heterogeneity.

## 3. Results

### 3.1. Search Results

**Figure 1:**
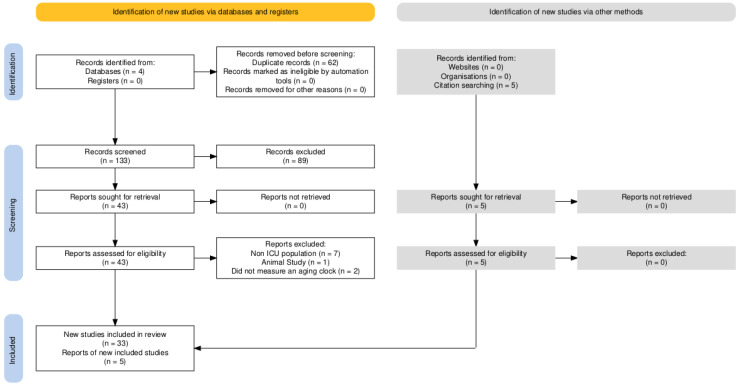
PRISMA-ScR flow diagram illustrating the study selection process for this scoping review.

The database search identified 195 records across PubMed/MEDLINE, Scopus, Web of Science, and Embase. After removing 62 duplicate entries, 133 records remained for title and abstract screening. Of these, 89 articles were excluded because they did not involve critically ill adult patients, did not evaluate a biological aging biomarker, or were non-original publications. A total of 43 full-text articles were assessed for eligibility, resulting in the exclusion of 10 studies for reasons including non-ICU population (n = 8), animal study (n = 1), and failure to measure a deep aging clock (n = 2). In addition to database searching, backward citation screening of included articles was performed, identifying additional eligible studies (n = 5), thereby increasing the total number of included articles to 38. Ultimately, 38 original studies met the inclusion criteria and were incorporated into the final synthesis. The complete screening process is depicted in Figure 1.

### 3.2. Characteristics

The final set of 38 included studies encompassed a broad spectrum of critically ill adult populations and clinical contexts. Sample sizes varied considerably, ranging from small mechanistic or biomarker-focused cohorts of fewer than 30 participants to large single or multicenter studies enrolling more than 2000 patients. The studies collectively represented a wide range of ICU pathologies.

A diverse array of biological specimens was used across studies, most commonly whole blood, plasma, serum, peripheral blood mononuclear cells (PMBCs), leukocyte fractions, and cfDNA. The timing of biomarker acquisition also varied; most studies collected samples at ICU admission. In contrast, others used serial sampling throughout the ICU stay or at specific time points during critical illness.

Across all studies, the most frequently reported clinical outcomes included ICU and hospital mortality, organ failure severity, length of stay, ventilator-free days, and longer-term functional recovery. Taken together, the included studies demonstrate substantial heterogeneity across patient populations, biomarker modalities, sampling methods, and clinical endpoints, reflecting the emerging, multidimensional nature of deep aging clock research in critical care. Taken together, these findings highlight marked heterogeneity in patient populations, biomarker modalities, sampling strategies, and clinical endpoints, underscoring both the complexity of studying biological aging in critical illness and the need for integrative, cross-domain synthesis to identify convergent prognostic signals.

### 3.3. Clock Classification

To synthesize heterogeneous aging metrics across critical care studies, we categorized all biological clocks and aging markers identified in the reviewed literature by their underlying biological domain and analytical framework. These clocks span epigenetic DNA methylation-based estimators, telomere and senescence markers, cfDNA-derived signals, proteomic and metabolomic networks, transcriptomic programs, and phenotypic laboratory-based indices. This domain-based organization is intended not merely to catalog biomarkers, but to facilitate cross-domain comparison and integrative interpretation of convergent biological signals in critical illness. The classification in Table 1 below provides a structured overview of the aging clocks evaluated in ICU populations and highlights the breadth of biological processes captured by current aging biomarkers in critical illness.

## 4. Discussion

### 4.1. General ICU Trends of Biological Clocks

Despite their methodological diversity, many biological aging clocks appear to converge on shared pathophysiological processes central to critical illness. Across epigenetic, proteomic, metabolomic, and cfDNA-based measures, consistent associations emerge with systemic inflammation, immune dysregulation, cellular senescence, mitochondrial stress, endothelial dysfunction, and impaired tissue repair. This convergence suggests that different clock modalities may capture complementary facets of a common stress-induced biological aging response, rather than unrelated phenomena. From this perspective, biological aging in critical illness should be viewed as a multidimensional systems-level process, with different biomarkers reflecting distinct but interconnected layers of the same underlying pathobiology.

In critically ill populations, biological aging markers consistently capture vulnerability and loss of physiological reserve that are not reflected by chronological age or standard ICU severity scores. Epigenetic clocks, telomere-related measures, and multi-omic biomarkers indicate that critical illness is often accompanied by biological age acceleration, which is independently associated with mortality, organ dysfunction, and impaired recovery. These signals reflect the combined impact of baseline frailty and acute inflammatory stress, positioning biological clocks as dynamic indicators rather than static aging measures. Collectively, current evidence suggests that biological aging metrics provide mechanistic and prognostic insight into resilience and recovery capacity in the ICU.

#### 4.1.1. Biological Age Acceleration and In-Hospital Mortality in Critically Ill Patients

Three retrospective cohort studies conducted in a tertiary intensive care unit in Western Australia consistently demonstrated a robust association between accelerated biological aging and hospital mortality, as quantified by PhenoAge-based metrics [11,12,13]. Across these cohorts, biological age acceleration was a stronger and more consistent predictor of adverse outcomes than chronological age, with prognostic value that persisted after adjustment for comorbidity burden and established ICU severity scores.

In the first cohort of 877 critically ill patients [11], with an overall hospital mortality of 10.6%, both PhenoAge residuals and a frailty index were independently associated with higher mortality after adjusting for chronological age, diabetes mellitus, and APACHE II score, indicating that biological age captures prognostic information not fully explained by acute illness severity or traditional demographic factors [11]. Similarly, in a second cohort [12] of 1073 patients, biological age and frailty were modestly but significantly correlated, whereas chronological age did not differ between survivors and non-survivors. In contrast, both crude PhenoAge values and regression-derived PhenoAge residuals were substantially higher among non-survivors and independently predicted hospital mortality. In adjusted survival analyses, PhenoAgeAccel remained significantly associated with mortality alongside the Clinical Frailty Scale, even after accounting for comorbidities, diabetes mellitus, chronological age, and elevated APACHE II scores, reinforcing the independent prognostic contribution of biological aging metrics in the ICU setting [12].

These findings were further reinforced in a larger cohort [13] of 2950 ICU patients, of whom 9.9% died before hospital discharge. In this analysis, being biologically older than one’s chronological age was associated with an almost twofold increase in unadjusted mortality risk, with a clear dose–response relationship that did not plateau until the biological-chronological age gap exceeded approximately 20 years. Importantly, this association remained statistically significant after adjustment for comorbid conditions and acute illness severity, underscoring the robustness of biological age acceleration as a mortality predictor across a broad ICU population [13]. Accelerated biological aging was more prevalent among patients with chronic cardiovascular disease, end-stage renal failure, cirrhosis, immune-mediated disease, diabetes mellitus, or exposure to immunosuppressive therapy; however, these factors did not fully account for the observed excess mortality risk [13].

Taken together, these three studies converge on the conclusion that biological age, as estimated by PhenoAge-based algorithms, consistently outperforms chronological age in mortality prediction among critically ill patients and retains independent prognostic value beyond conventional ICU risk stratification tools such as APACHE II [11,12,13].

#### 4.1.2. Associations Between Biological Aging, Adiposity, and Frailty in Critically Ill Patients

Beyond hospital mortality, two of the retrospective cohorts mentioned above [11,12] examined how biological age relates to frailty and BMI.

In the 877-patient cohort [11], Body Mass Index (BMI) showed a significant U-shaped association with both frailty (*p* = 0.003) and PhenoAge residuals (*p* = 0.001), indicating higher biological age acceleration and frailty among underweight and morbidly obese patients. However, BMI was not associated with mortality (*p* = 0.0388), suggesting that body composition may influence outcomes indirectly through biological aging and frailty rather than as an independent risk factor. By contrast, PhenoAgeAccel was present in 46.8% of patients in this cohort and was significantly associated with mortality (*p* = 0.001). These findings suggest that biological age may be a more informative metric for capturing vulnerability than BMI [11].

In the 1073-patient cohort [12], both PhenoAge (AUROC 0.622, 95% CI 0.568–0.676) and its residuals (AUROC 0.627, 95% CI 0.573–0.680) performed comparably to the Clinical Frailty Scale (CFS) (AUROC 0.601, 95% CI 0.544–0.658) in predicting hospital mortality. This supports the interpretation of frailty as a clinical phenotype of biological aging [12].

#### 4.1.3. Biological Age and ICU Readmission Association

Kwok et al. [14] extended the evaluation of biological age to unplanned ICU readmission, a patient-centered outcome reflecting post-ICU vulnerability. Among 2950 patients, 153 (5.2%) experienced unplanned ICU readmission during the same hospitalization. Patients who were readmitted were more likely to exhibit accelerated biological aging, with PhenoAgeAccel present in 52% of readmitted patients compared with 43% of those not readmitted (52% vs. 43%, *p* = 0.031), and higher PhenoAge residuals in the readmission group (*p* = 0.005) [14]. In contrast, chronological age, APACHE II score, and the use of mechanical ventilation, vasopressors, or renal replacement therapy during the ICU admission did not differ significantly between those with and without subsequent ICU readmission [14].

The findings support the interpretation that biological age reflects reduced physiological reserve and impaired recovery capacity rather than acute illness severity alone. The absence of associations between readmission and conventional ICU severity indicators further suggests that biological age captures an underlying vulnerability that influences post-ICU outcomes beyond traditional risk stratification measures.

#### 4.1.4. Epigenetic Markers in Critical Illness

Beyond epigenetic aging clocks derived from clinical and molecular parameters, biological aging in critical illness has also been examined using TL as a potential biomarker of physiological stress and vulnerability. In a prospective observational study of 40 critically ill patients, TL dynamics were assessed during the ICU stay [15]. Marked interindividual heterogeneity in TL changes was observed over a mean interval of 7.2 ± 2.5 days, with telomere shortening in 21 patients, lengthening in 11, and no significant change in 8. Patients with telomere shortening were significantly younger than those with lengthening (45.4 vs. 61.5 years, *p* < 0.023). Changes in TL correlated with changes in white blood cell counts, with shortening associated with declining leukocyte levels. A trend toward shortening was observed in patients with sepsis, though this did not reach statistical significance (*p* = 0.07) [15]. No significant associations were identified between TL changes and clinical outcomes, indicating limited prognostic value of short-term telomere dynamics in the ICU setting. This conclusion is further constrained by the small sample size (n = 40) [15].

Beyond blood-based aging measures, Van Dyck et al. [16] investigated skeletal muscle DNA methylation in 172 critically ill patients and 20 healthy controls. Muscle biopsies obtained on ICU Day 8 ± 1 revealed 565 differentially methylated CpG sites (q < 0.05, corresponding to *p* < 0.00005), of which 75.6% were hypomethylated in ICU patients. Mean absolute methylation differences averaged 3.2% (SEM 0.07%), with individual loci reaching up to 16.9% [16].

The methylome analysis revealed differential methylation across approximately 400 genes involved in muscle integrity, mitochondrial metabolism, and neuromuscular signaling. Two notable hypomethylated promoter regions were identified in *HIC1*, a transcriptional regulator linked to muscle regeneration, and *NADK2*, a gene central to mitochondrial NADP biosynthesis [16]. Notably, 90% of differentially methylated genes demonstrated concordant expression changes, and targeted analysis of 10 genes validated significant differential transcription with effect sizes from 18.3% to 77.5%. These findings underscore a coordinated epigenetic reprogramming of muscle-related pathways in critical illness [16].

#### 4.1.5. Proteomics and Progression of Chronic Diseases in ICU Patients

In an observational study conducted in China [17], the authors investigated whether a molecular age (MA) metric derived from routine laboratory biomarkers could reflect the development and progression of chronic disease. The analysis focused on biochemical tests and complete blood counts from ICU patients who died within one month of admission, along with multiple comparator populations spanning health, chronic disease, and age extremes [17].

Six cohorts were analyzed: a young reference group (n = 197; 20–30 years), an elderly group (n = 198; >80 years), an ICU cohort of critically ill patients (n = 106; mean age 75.6 ± 8.1 years), a healthy middle-aged group aged 55–75 years (n = 20; 10 females, 10 males), a disease cohort including cancer, coronary or cerebrovascular disease, and Chronic obstructive pulmonary disease (COPD), and a large population control group aged 50–80 years (n = 721; mean age 65.0 ± 10.3). The disease cohort comprised 274 cancer patients (mean age 63.2 ± 8.1), 144 with coronary or cerebrovascular disease (64.9 ± 8.4), and 165 with COPD (67.7 ± 13.7) [17]. Among all evaluated biomarkers, albumin was identified as the only stable and valid marker for estimating MA. MA was defined using a survival-based model incorporating albumin and chronological age: MA score = 0.02 × albumin − 0.01 × age + 0.45 [17].

An MA score < 0.5 was interpreted as indicating a pre-chronic disease stage (PCDS) at the molecular level. This threshold was supported by the observation that 94.3% of ICU patients with chronic disease had an MA score < 0.5, compared with only 5.1% of healthy elderly individuals. MA scores were significantly lower across all disease groups compared with controls, and the proportion of individuals classified as PCDS (MA < 0.5) was higher in all disease groups than in the elderly group, despite similar chronological ages [17].

Overall, the study [17] shows that end-of-life ICU patients have markedly different proteomic profiles compared with healthy elderly and younger populations, supporting the idea that MA derived from routine laboratory data may provide insight into biological aging and health status beyond chronological age alone.

### 4.2. Sepsis

Over the past decade, attention has increasingly focused on epigenetic alterations in critically ill septic patients, particularly DNA methylation changes observed in sepsis. Beyond their diagnostic or prognostic utility, these studies provide important mechanistic insight into how acute inflammatory stress induces coordinated, non-random methylomic remodeling—a necessary condition for biological aging estimates derived from deep DNA methylation clocks. Early studies provided foundational evidence that sepsis induces reproducible and biologically meaningful methylation remodeling in whole blood cells.

The first study to demonstrate that a septic episode can induce DNA-methylation alterations in human monocytes—particularly those associated with the acquisition of a tolerized, hyporesponsive immune phenotype—was conducted in Spain [18]. Monocytes isolated within the first 12 h of sepsis diagnosis from 14 patients (mean age 74.6 ± 14.5 years; SOFA 3.9 ± 2.0) were compared with those from 11 healthy controls (mean age 51 ± 11.8 years). The study revealed broad, pathway-level remodeling of DNA methylation. Hypermethylated CpG sites were enriched in innate immune signaling cascades, including Mitogen-Activated Protein Kinase (MAPK) and Nuclear Factor Kappa-Light-Chain-Enhancer of Activated B Cells (NF-κB) pathways and cytokine-chemokine signaling networks, whereas hypomethylated CpGs clustered within genes central to monocyte function, including interferon-γ signaling and MHC class II expression [18].

Importantly, DNA methylation shifts correlated strongly with circulating IL-6 (r > 0.5; Δβ ≥ 0.1) and IL-10 (r > 0.5; Δβ ≥ 0.15) levels, key mediators of systemic inflammation and immunosuppression. Additional hypermethylation in the Wnt signaling pathway, together with enrichment of Activator protein 1 (AP-1) and Signal transducer and activator of transcription (STAT) factor motifs, further supported inflammation-driven regulatory remodeling. Notably, methylation changes scaled with organ dysfunction: higher SOFA scores were associated with progressively larger methylation differences (*p* < 0.01; r > 0.6), suggesting a dose–response relationship between physiological stress and epigenetic state [18].

Further support for this concept was provided by a subsequent Spanish study [19], which examined leukocyte DNA methylation patterns in septic shock, sepsis, and critically ill non-septic patients. Blood samples collected in the first 24 h of sepsis diagnosis across discovery (n = 12) and validation (n = 30) cohorts showed tens of thousands of Differentially Methylated Positions (DMPs), particularly in septic shock (38,276 hypermethylated and 30,462 hypomethylated). The DNA methylation patterns of the 6657 DMPs with a False Discovery Rate (FDR) < 0.01 identified in the septic shock vs. critically ill patients comparison effectively classified patient groups based on their SOFA score. Furthermore, the results showed a strong association between DNA methylation changes and lactate levels, indicating a direct relationship between DNA methylation patterns and clinical phenotype severity [19].

Two distinct analytical approaches identified 1256 Differentially Methylated Region (DMRs) in septic shock patients compared with critically ill patients (458 hypermethylated and 798 hypomethylated) [19]. DMRs were enriched in genes regulating immune activation, cytokine signaling, and immunosuppression, including hypomethylation of IL10 and S100A8. Collectively, these findings suggest a complex epigenetic landscape marked by concurrent signatures of immune hyperactivation and exhaustion. While some methylation changes may be induced by sepsis itself, the data also support a role for pre-existing epigenetic states in determining progression from sepsis to septic shock, consistent with inter-individual variability in biological aging and immune reserve [19].

Larger whole-blood studies have reinforced and extended these observations. A multicenter investigation from Canada and Portugal [20] analyzed DNA methylation profiles from septic (n = 66) and non-septic (n = 68) critically ill patients on the first day of ICU admission. A total of 668 DMRs corresponding to 443 genes distinguished septic from non-septic patients. Notably, disease-association analyses revealed substantial overlap with autoimmune-related gene networks, particularly IFN-regulated genes, whereas comparative analyses suggested that certain methylation signatures were specific to sepsis [20].

Functional enrichment analyses identified altered activity in methyltransferase pathways, antigen presentation, and cell adhesion. Weighted gene coexpression network analysis further identified modules associated with illness severity, vasopressor requirement, and ICU length of stay, underscoring the relevance of coordinated epigenetic remodeling to clinical trajectories rather than isolated disease classification [20].

A separate study using the same cohort of patients [21] evaluated epigenetic age acceleration in 134 ICU patients with sepsis (n = 66) and without (n = 68) using both the Hannum and PhenoAge clocks. Septic patients demonstrated significant epigenetic age acceleration using the PhenoAge clock (+4.97 years, *p* = 0.045) compared with non-septic patients. The Hannum clock showed no significant acceleration in either group (*p* = 0.07). Regarding mortality, non-survivors exhibited significant PhenoAge acceleration (+7.63 years, *p* = 0.004) compared with survivors. Again, the Hannum clock showed no significant association with survival status (*p* = 0.102). Although the two cohorts did not differ quantitatively in the number of comorbidities, they may have differed qualitatively. These findings suggest clock-specific sensitivity, with PhenoAge potentially capturing inflammation and immune-related processes relevant to critical illness outcomes [21].

**Figure 2:**
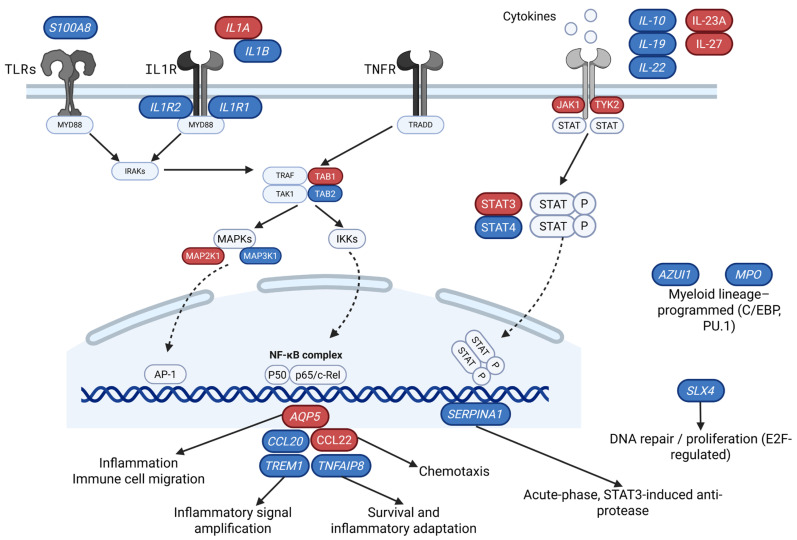
Immune Signaling Pathways Altered in Sepsis. Schematic representation of innate immune and cytokine signaling pathways commonly dysregulated in sepsis, including TLR, IL-1R, TNFR, MAPK, NF-κB, and JAK–STAT signaling. Selected molecules encoded by genes exhibiting differential DNA methylation in the referenced studies are highlighted (red, hypermethylation; blue, hypomethylation), emphasizing epigenetic modulation of inflammatory signaling. Created in https://BioRender.com. Abbreviations: TLRs—Toll-like receptors; IL1A—interleukin-1 alpha; IL1B—interleukin-1 beta; IL1R—interleukin-1 receptor; IL1R1—interleukin-1 receptor type 1; IL1R2—interleukin-1 receptor type 2; MYD88—myeloid differentiation primary response 88; IRAKs—interleukin-1 receptor–associated kinases; TNFR—tumor necrosis factor receptor; TRADD—TNF receptor–associated death domain; TRAF—TNF receptor–associated factor; TAB1—TAK1-binding protein 1; TAB2—TAK1-binding protein 2; TAK1—transforming growth factor-β–activated kinase 1; MAPKs—mitogen-activated protein kinases; MAP2K1—mitogen-activated protein kinase kinase 1 (MEK1); MAP3K1—mitogen-activated protein kinase kinase kinase 1; IKKs—IκB kinases; NF-κB—nuclear factor kappa-light-chain-enhancer of activated B cells; AP-1—activator protein-1; JAK1—Janus kinase 1; TYK2—tyrosine kinase 2; STAT—signal transducer and activator of transcription; STAT3—signal transducer and activator of transcription 3; STAT4—signal transducer and activator of transcription 4; IL-10—interleukin-10; IL-19—interleukin-19; IL-22—interleukin-22; IL-23A—interleukin-23 subunit alpha; IL-27—interleukin-27; AQP5—aquaporin 5; CCL20—C-C motif chemokine ligand 20; CCL22—C-C motif chemokine ligand 22; TREM1—triggering receptor expressed on myeloid cells 1; TNFAIP8—tumor necrosis factor alpha–induced protein 8; SERPINA1—serpin family A member 1; AZU1—azurocidin 1; MPO—myeloperoxidase; C/EBP—CCAAT/enhancer-binding protein; PU.1—PU.1 transcription factor (SPI1); SLX4—SLX4 structure-specific endonuclease subunit.

Building on this groundwork, López-Cruz et al. [22] conducted a prospective case–control study evaluating leukocyte DNA methylation profiles in community-acquired sepsis versus infection-matched controls. The discovery cohort comprised 16 sepsis cases and 16 controls, with validation in an independent cohort of 28 adults (14 sepsis, 14 controls; median age 78, IQR 68–84). Four immune-related genes—SERPINA1, AZU1, MPO, and SLX4—showed robust, reproducible hypomethylation across both cohorts [22]. Gene-specific methylation levels correlated strongly with admission SOFA scores and early procalcitonin concentrations. At the same time, associations with CD4^+^ T-cell counts, especially for AZU1, suggested links between epigenetic state and immune composition or activation state. When assessed against Quick Sequential Organ Failure Assessment (qSOFA), SOFA, and APACHE II, methylation markers—particularly SLX4 and MPO (Area Under the Curve [AUC] 0.821 and 0.801, respectively)—provided modest improvements in predicting poor prognosis. At the same time, SERPINA1 hypomethylation exhibited strong diagnostic potential for sepsis (AUC 0.858) [22]. Although modest improvements over clinical scores for prognostic discrimination were observed, their primary relevance lies in demonstrating that acute immune-related methylation changes encode information about physiological reserve and system-level failure—features central to aging clock interpretation rather than disease diagnosis alone [22]. Figure 2 schematically integrates the sepsis-associated biological aging pathways identified in this review.

Complementary evidence for prognostic epigenetic regulation came from a German cohort study examining methylation of an NF-κB-binding site in the aquaporin-5 (AQP5) promoter [23]. Whole blood samples were collected from 135 patients within 24 h of sepsis diagnosis, including 88 survivors and 47 non-survivors. A higher methylation level at cytosine site nt-937 in the AQP5 promoter, linked to NF-κB binding, was observed in non-survivors compared with survivors (*p* = 0.002, padj = 0.014). Furthermore, CpG methylation at this promoter position in septic patients was associated with substantially higher 30-day mortality (HR: 3.31; 95% CI: 1.54–6.23; *p* = 0.002), making it an important and independent prognostic factor. Although single-locus associations differ conceptually from multi-CpG aging clock architectures, such sites may represent pathway hubs that disproportionately influence clock outputs during acute inflammatory stress [23].

Beyond DNA methylation changes, sepsis patients had significantly shorter Peripheral Blood Leukocyte Telomere Length (PBL-TL) than non-septic critically ill patients (*p* = 0.0007) [24]. Shorter telomeres were independently associated with worse 90-day survival (adjusted HR 1.5; 95% CI 1.2–2.0 per 1 kb decrease; *p* = 0.001) and 1-year survival (adjusted HR 1.4; 95% CI 1.1–1.8; *p* = 0.003). These findings were replicated in an independent sepsis validation cohort, in which the median PBL-TL was 6.25 kb (IQR 5.87–6.58 kb). In this cohort, shorter PBL-TL was associated with worse 60-day survival (adjusted HR 1.6; 95% CI 1.2–2.1 per 1 kb decrease; *p* = 0.003) and a markedly increased risk of severe ARDS (adjusted OR 2.5; 95% CI 1.1–6.3 per 1 kb decrease; *p* = 0.044). Collectively, these results indicate that advanced MA, as indexed by telomere attrition, contributes meaningfully to adverse outcomes in critical illness and sepsis [24].

Importantly, the association between telomere shortening and sepsis outcomes is further supported by genetic evidence. Xu et al. [25] employed a bidirectional Mendelian randomization framework to investigate the causal relationship between leukocyte telomere length (LTL) and sepsis susceptibility. Using summary statistics from genome-wide association studies (GWAS) encompassing 472,174 individuals, single-nucleotide polymorphisms associated with LTL were selected as instrumental variables [25].

The analysis [25] demonstrated a significant association between genetically predicted shorter LTL and increased risk of sepsis (*p* = 0.008). Specifically, a one standard deviation decrease in genetically predicted LTL was associated with a 1.161-fold increase in sepsis risk (OR 1.161; 95% CI 1.039–1.297). A similar estimate was obtained using alternative Mendelian randomization methods, yielding an odds ratio of 1.254 (95% CI 1.045–1.505; *p* = 0.016). These findings provide evidence that shortened telomeres are not merely a consequence of critical illness but may causally predispose individuals to sepsis, reinforcing the concept that biological aging influences vulnerability to severe infection [25].

Studies of circulating cfDNA provide a complementary and dynamic perspective on molecular aging and tissue injury in sepsis. Cano-Gamez et al. [26] investigated the cfDNA landscape in plasma samples from 46 patients admitted with sepsis (86 samples collected longitudinally) and 14 healthy controls. cfDNA concentrations were dramatically elevated in patients with sepsis compared with controls, with an average 41.2-fold increase. Among septic patients, those admitted to the ICU had approximately 10-fold higher cfDNA concentrations than those managed in the emergency department or general medical ward [26].

Furthermore, cfDNA levels increased progressively with escalating organ support requirements, including mechanical ventilation, vasopressor therapy, and inotropic support, indicating a close relationship between cfDNA burden and disease severity [26]. Notably, these increases occurred despite significant compositional differences in cfDNA fragment origin, suggesting that impaired hepatic clearance during sepsis rather than altered tissue contribution, was a primary driver of cfDNA accumulation [26].

Beyond concentration, cfDNA methylation patterns revealed that methylation changes at specific genomic regions accompanied fluctuations in organ function. Simultaneously, time since ICU admission exerted a substantial influence on the cfDNA methylomic profile [26]. These observations indicate that cfDNA methylation captures dynamic disease trajectories rather than static disease states. Additionally, nucleosome footprinting analyses demonstrated that cfDNA retains information about tissue-specific gene activity, providing a potential window into real-time regulation across injured organs [26].

Collectively, evidence from DNA methylation, TL, and circulating cfDNA profiling indicates that critical illness, particularly sepsis, induces a multidimensional shift in molecular aging markers that reflects both baseline biological vulnerability and acute, stress-induced biological age acceleration. Across studies, sepsis is consistently associated with structured, severity-dependent DNA methylation remodeling in circulating blood cells, with changes that are non-random and closely aligned with immune dysfunction, organ failure, and mortality. These convergent findings support the interpretation that sepsis is accompanied by impaired immune resilience and accelerated biological aging, while also highlighting critical methodological limitations, including limited longitudinal sampling, predominantly single-center designs, and incomplete adjustment for leukocyte heterogeneity when interpreting deep aging clocks in the ICU population.

### 4.3. Acute Respiratory Distress Syndrome

In a multicenter observational study conducted across four hospitals in China [27], the authors applied an integrative proteomic and metabolomic approach to delineate host-response networks in ARDS and identify early prognostic biomarkers [27]. The study included a discovery cohort of 130 ICU patients with ARDS and an independent multicenter validation cohort of 183 patients with ARDS. Clinical comparisons confirmed that non-survivors exhibited greater disease severity, including lower PaO_2_/FiO_2_ ratios and albumin levels and higher SOFA scores.

High-throughput serum proteomics quantified 2669 high-confidence proteins, revealing progressive molecular divergence from healthy controls to disease controls and ultimately to ARDS. Differential abundance analysis identified 214 proteins consistently dysregulated in ARDS, the vast majority of which were upregulated. Network-based pathway enrichment demonstrated coordinated activation of oxidative phosphorylation, Vascular Endothelial Growth Factor (VEGF) signaling, MAPK, JAK–STAT, NF-κB, and PI3K–Akt pathways, indicating widespread metabolic reprogramming, inflammatory amplification, and endothelial dysfunction. Within the protein–protein interaction network, Mitogen-Activated Protein Kinase Kinase 1 (MAP2K1) emerged as a central hub, linking angiogenic, inflammatory, and stress-response signaling modules and highlighting kinase-driven pathway integration as a core feature of ARDS biology [27].

Parallel metabolomic profiling identified 214 differentially abundant metabolites, with prominent depletion of sphingolipid pathway components, particularly sphingosine-1-phosphate (S1P) and lysophosphatidylcholine (LPC) species. Integrated proteomic–metabolomic network analysis revealed the sphingolipid signaling pathway as the dominant cross-talk axis linking metabolic alterations to protein signaling networks. Reduced S1P levels correlated with higher SOFA scores and intersected with MAPK-mediated inflammatory and apoptotic pathways, including upregulation of BCL–2–associated X Protein (BAX) and BH3-interacting domain Death Agonist (BID), thereby linking lipid signaling imbalance to endothelial injury, immune dysregulation, and programmed cell death [27].

Using this network-informed framework, the authors identified 40 proteins associated with ARDS mortality and refined them into an 8-protein prognostic panel using machine-learning modeling. The final panel consisted of Vascular Cell Adhesion Molecule-1 (VCAM1), Lactate Dehydrogenase B (LDHB), Moesin, Filaggrin-2 (FLG2), LMNA (Lamin A/C), and Lipopolysaccharide-Binding Protein (LBP), which are upregulated in non-survivors, and downregulated TAGLN2 and Mannose-Binding Lectin 2 (MBL2). This model demonstrated strong predictive performance for ARDS mortality, achieving AUCs of 0.893 in the discovery cohort and 0.802 in the validation cohort, outperforming conventional clinical indices such as the SOFA score and PaO_2_/FiO_2_ ratio [27].

In a multicenter observational study conducted across three academic centers in the United States [28], the authors applied a multi-omics framework integrating transcriptomics, DNA methylation, proteomics, and genome-wide association data to identify early and intermediate biomarkers associated with ARDS mortality [28]. The primary objective was to uncover molecular pathways and biomarkers predictive of survival while advancing mechanistic understanding of ARDS pathogenesis.

The study [28] enrolled 568 adult ICU patients diagnosed with ARDS (mean age 52.5 ± 15.6 years; 54.9% male), with a 28-day mortality rate of 27%. Multi-omic profiling was performed across overlapping patient subsets and included genome-wide genotyping, DNA methylation analysis, RNA transcription profiling, and targeted plasma proteomics. Plasma protein analysis was conducted in 240 patients and focused on 11 candidate proteins selected for their known roles in inflammation, endothelial dysfunction, and lung injury, including angiopoietin-2 (Ang-2), IL-1β, interleukin-1 receptor type 2 (IL-1R2), IL-6, IL-8, VEGF, macrophage migration inhibitory factor (MIF), sphingosine-1-phosphate receptor 3 (S1PR3), receptor for advanced glycation end products (RAGE), high-mobility group box 1 (HMGB1), and extracellular nicotinamide phosphoribosyltransferase (eNAMPT) [28].

**Figure 3:**
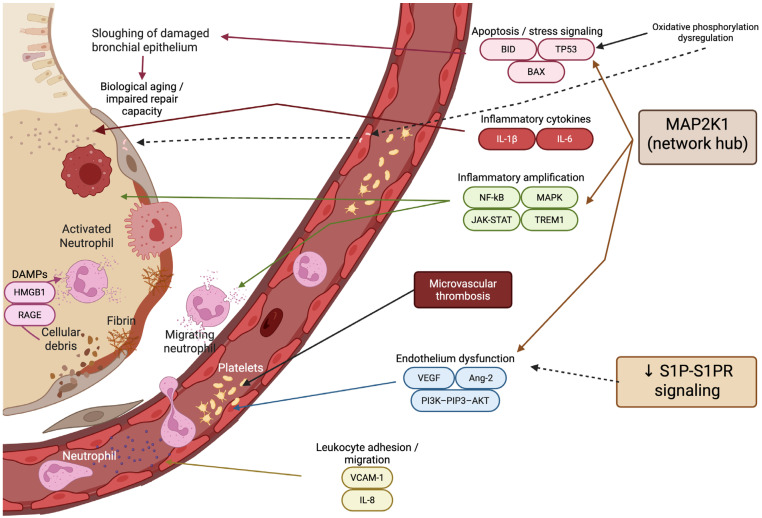
Network-level Dysregulation of Inflammatory, Endothelial, and Stress-Response Pathways in ARDS. Scheme depicting coordinated molecular and cellular processes driving ARDS. Inflammatory amplification via NF-κB, MAPK, JAK–STAT, and TREM1 signaling is coupled with endothelial dysfunction involving VEGF, Ang-2, VCAM-1, and PI3K–PIP3–AKT pathways. MAP2K1 acts as a central hub linking inflammatory, angiogenic, and stress-response networks. Metabolic dysregulation, including impaired oxidative phosphorylation, converges on TP53-mediated apoptosis, while reduced S1P–S1PR signaling exacerbates endothelial barrier failure, together defining ARDS as a network-level disorder underlying mortality risk. Created in https://BioRender.com. Abbreviations: DAMPs—damage-associated molecular patterns; HMGB1—high mobility group box 1; RAGE—receptor for advanced glycation end products; IL-1β—interleukin-1 beta; IL-6—interleukin-6; NF-κB—nuclear factor kappa-light-chain-enhancer of activated B cells; MAPK—mitogen-activated protein kinase; MAP2K1—mitogen-activated protein kinase kinase 1 (MEK1); JAK-STAT—Janus kinase–signal transducer and activator of transcription signaling pathway; TREM1—triggering receptor expressed on myeloid cells 1; BID—BH3-interacting domain death agonist; BAX—BCL2-associated X protein; TP53—tumor protein p53; VEGF—vascular endothelial growth factor; Ang-2—angiopoietin-2; PI3K—phosphoinositide 3-kinase; PIP3—phosphatidylinositol (3,4,5)-trisphosphate; AKT—protein kinase B; VCAM-1—vascular cell adhesion molecule-1; IL-8—interleukin-8; S1P—sphingosine-1-phosphate; S1PR—sphingosine-1-phosphate receptor.

From a proteomic perspective, Ang-2 emerged as the most robust protein biomarker associated with ARDS mortality. Elevated Ang-2 levels on day 7 were significantly related to mortality, with levels decreasing in survivors but remaining persistently elevated in non-survivors. Ang-2 alone showed moderate prognostic performance (AUC 0.71; 90% CI 0.51–0.91), outperforming other measured proteins and clinical protein combinations. IL-1R2 showed weaker predictive value, and the baseline protein panel demonstrated limited discrimination, underscoring the temporal importance of dynamic endothelial injury markers rather than static early measurements [28]. Figure 3 depicts the proteomic network of ARDS as covered in this section.

At the network level, transcriptomic analysis identified a 9-gene expression signature (TNP01, NUP214, HDAC1, HNRNPA1, GATAD2A, FOSB, DDX17, PHF20, CREBBP) that differentiated survivors from non-survivors [28]. Pathway enrichment revealed coordinated activation of transcriptional regulation networks rather than isolated gene effects. The most significant pathway was TP53-regulated transcription (adjusted *p* = 6.84 × 10^−20^), with expression peaking at day 14 in non-survivors before returning toward baseline. Additional enriched pathways included RNA polymerase II transcription, PIP3–AKT signaling, and intracellular second messenger signaling, collectively reflecting sustained cellular stress responses, impaired repair mechanisms, and dysregulated apoptosis in fatal ARDS [28].

DNA methylation analysis identified two probes (F-Box Protein 6-FBXO6 and MAP3K14) with suggestive associations with mortality. In contrast, GWAS and RNA sequencing analyses failed to identify genome-wide significant variants or robust differentially expressed genes, underscoring that mortality risk was more strongly reflected at the pathway and network levels than at single genetic loci. A logistic regression model based on the 9-gene transcriptional signature achieved an AUC of 0.83 (90% CI 0.62–1.00), supporting the utility of coordinated transcriptional networks for prognostication [28].

When compared to the Chinese integrative proteomic–metabolomic ARDS study by Lin et al. [27], significant conceptual parallels and distinctions emerge. Both studies converge on endothelial dysfunction and inflammatory network activation as central drivers of ARDS mortality. Ang-2, identified here [28] as a key prognostic protein, aligns mechanistically with the VEGF and MAPK signaling hubs highlighted by Lin et al. [27], reinforcing endothelial barrier disruption as a core pathogenic axis. However, whereas Lin et al. relied on a targeted protein panel and transcription-centric pathway inference, this study [28] expanded network resolution through unbiased serum proteomics and metabolomics, identifying the sphingolipid-MAPK signaling axis and oxidative phosphorylation as dominant cross-talk pathways.

In contrast to Lin et al.’s [27] eight-protein prognostic panel embedded within a protein–protein interaction network, this study demonstrated that dynamic endothelial injury markers (Ang-2) and stress-response transcriptional programs (TP53-centered networks) carry prognostic significance even in the absence of strong single-gene or single-base-pair effects. Together, these studies suggest an evolution in ARDS biomarker research—from pathway-informed candidate approaches toward fully network-resolved, multi-omic prognostic models—while consistently implicating endothelial dysfunction, inflammatory amplification, and impaired cellular repair as shared molecular substrates of ARDS mortality.

DNA methylation alterations have also been explored in ARDS. Zhang et al. [29] analyzed publicly available GEO datasets comprising 99 patients with ARDS within 48 h of onset and 136 healthy controls. Thirty methylation alterations were identified, four of which demonstrated near-perfect discriminatory performance (AUC = 0.99). The four highly discriminatory CpG loci mapped to genomic regions linked to immune signaling and cell migration (PTPRN2), endothelial and epithelial regulation (LINC00599), cytoskeletal organization and cell–cell interactions (FARP1), and stress-response signaling (intergenic MAPK-regulatory region), and pathway enrichment implicated mTOR- and MAPK-mediated responses rather than dominant single-gene effects [29].

Additionally, Liu et al. [24] conducted an extensive prospective observational study measuring PBL-TL in 937 critically ill patients with and without ARDS, with an independent validation cohort of 394 patients with sepsis. DNA was isolated from leukocytes collected within 24 h of ICU admission. In the primary cohort, the median PBL-TL was 6.96 kb (IQR 6.47–7.41 kb), and shorter TL was independently associated with worse 90-day and 1-year survival across all critically ill patients [24].

Specifically, for every 1 kb decrease in PBL-TL, the adjusted hazard ratio for 90-day mortality was 1.3 (95% CI 1.1–1.5; *p* = 0.004), with an identical effect size observed for 1-year mortality (adjusted HR 1.3; 95% CI 1.1–1.5; *p* = 0.004). After adjustment for relevant covariates, the 90-day mortality rate increased by 36% per 1 kb decrease in TL. Importantly, while PBL-TL did not differ significantly between ARDS and non-ARDS patients overall and was not associated with ARDS development (*p* = 0.87), shorter telomeres were associated with an increased risk of developing severe ARDS (adjusted OR 1.7; 95% CI 1.2–2.5 per 1 kb decrease; *p* = 0.006) [24].

In summary, converging multi-omic evidence indicates that ARDS mortality is driven by coordinated, network-level disturbances involving endothelial injury, inflammatory amplification, metabolic reprogramming, and impaired cellular repair, rather than isolated molecular signals. Dynamic biomarkers, including Ang-2, stress-response transcriptional programs, epigenetic remodeling, and telomere shortening, capture both baseline biological vulnerability and acute biological age acceleration, consistently outperforming static clinical indices. Together, these findings support a transition toward integrative, biologically informed prognostic models that better stratify risk and guide precision approaches in ARDS.

### 4.4. COVID-19

The application of epigenetic clocks to adverse outcomes and disease progression has emerged as a rapidly expanding area of research, particularly during the COVID-19 pandemic, where marked heterogeneity in clinical outcomes was observed among patients with comparable disease severity scores [30]. This unexplained variability has highlighted the limitations of conventional clinical stratification. It suggests that biological age metrics derived from epigenetic clocks may capture latent vulnerability and resilience, thereby shedding light on differential trajectories and outcomes in COVID-19 patients beyond traditional measures of severity [31]. The observational studies summarized in this section employed multiple epigenetic aging algorithms, including the Horvath, Hannum, GrimAge, and PhenoAge clocks, to predict outcomes associated with SARS-CoV-2 infection, such as the need for intensive care unit (ICU) admission, mechanical ventilation, and mortality.

Andargie et al. [32] conducted an analysis using nuclear cfDNA, demonstrating that nuclear cfDNA showed high discriminative performance in distinguishing hospitalized patients requiring ICU admission from those who did not (*p* = 0.001) [32]. In addition, tissue-specific cfDNA profiles derived from monocytes, neutrophils, erythroblasts, vascular endothelium, hepatocytes, adipocytes, pancreas, bladder, intestine, kidney, heart, lung, and head and neck tissues further confirmed this association. In contrast, traditional inflammatory markers demonstrated inferior discriminatory performance compared with cfDNA with AUC of 0.582 for CRP (95% CI = 0.359–0.805; *p* = 0.4611), 0.689 for D-dimer (95% CI = 0.480–0.894; *p* = 0.099), 0.764 for neutrophil/lymphocyte ration (95% CI = 0.567–0.959; *p* = 0.0177), and 0.763 for troponin (95% CI = 0.550–0.976; *p* = 0.0343) [32].

Calzari et al. [33] reported that epigenetic signature analysis identified a 21-CpG epi-signature capable of distinguishing individuals with severe COVID-19 outcomes from COVID-19–negative subjects (*p* = 3.3 × 10^−10^), but not between patients with mild COVID-19 and COVID-19–negative controls (*p* = 0.11), suggesting that this epigenetic pattern is more closely associated with disease severity rather than viral presence itself [33]. Similarly, Corley et al. [34] applied the Horvath, GrimAge, and PhenoAge epigenetic clocks and demonstrated a significantly increased mortality risk in patients with severe COVID-19 compared with uninfected controls, as well as individuals with primary HIV infection and HIV/COVID-19 coinfection (*p* < 0.05). Using DNA methylation-based mortality biomarkers, including cystatin C and tissue inhibitor of metalloproteinase-1 (TIMP-1) as indicators of renal dysfunction and fibrosis, the authors further observed significantly higher methylation levels in severe COVID-19 patients relative to both uninfected controls and influenza patients (*p* < 0.05). Collectively, these findings suggest that epigenetic age acceleration in severe COVID-19 is not driven solely by viral infection but reflects host-related biological vulnerability linked to overall clinical status and disease severity [34].

Márquez-Salinas et al. [35] demonstrated that patients with positive PhenoAge acceleration (PhenoAgeAccel > 0) had a significantly higher risk of adverse outcomes, increased lethality, and impaired metabolic, respiratory, and immunologic function, even after adjustment for established COVID-19 risk factors, including sex and comorbidity burden [35].

From a longitudinal perspective, Bejaoui et al. [36] reported a significant decrease in epigenetic age acceleration among patients recovering from severe COVID-19 infection between ICU admission and the final pre-recovery time point, as measured by the Horvath clock (*p* = 0.0017), Hannum clock (*p* < 0.0001), and PhenoAge clock (*p* = 0.009) [36]. Additionally, Andargie et al. [32] reported that plasma nuclear cfDNA levels within the first two days of admission were 4.5-fold higher in patients who ultimately died from COVID-19 compared with those who recovered (*p* = 0.0001) [32]. Collectively, these findings suggest that epigenetic age and cfDNA measurements may have clinical utility for monitoring disease progression and identifying patients at risk for deterioration or death.

However, contrasting evidence has also been reported. Franzen et al. [37] demonstrated that although multiple epigenetic clocks correlated strongly with chronological age, including Horvath 2013 (*p* < 10^−300^), Horvath 2018 (*p* < 10^−283^), Hannum (*p* < 10^−284^), and Han (*p* < 10^−299^), no acceleration of epigenetic age was observed in analyzed COVID-19 blood samples, even after stratification by clinical outcome, including the presence or absence of ARDS [37].

TL has also been investigated as a marker of biological aging and disease severity in COVID-19 [38]. Viral infections induce upregulation of interferon-stimulated genes that regulate telomere-repeat-containing RNA (TERRA) transcription, potentially explaining the observed variability in TL responses to viral stressors [38].

Corley et al. [34] reported no significant decrease in DNA methylation-based TL in severe COVID-19 compared with uninfected controls (*p* = 0.22). However, they observed significantly shorter TL in individuals coinfected with HIV and mild-to-moderate COVID-19 compared with uninfected controls, influenza patients, and those with severe COVID-19 (*p* < 0.05) [34]. Bejaoui et al. [36] similarly reported no significant differences in TL between COVID-19 patients and healthy controls. However, longitudinal analysis revealed a considerable decrease in TL among deceased COVID-19 patients at the end of follow-up compared with baseline (*p* = 0.0015), confirmed by increased TL attrition acceleration (*p* = 0.0077). Notably, mixed linear modeling across continuous time points did not reveal overall differences between COVID-19 patients and healthy controls. Franzen et al. [37] similarly reported no significant telomere attrition in COVID-19 patients compared with healthy controls. However, their analysis was based on a small cohort of 19 patients and lacked adjustment for major risk factors.

Wang et al. [39] analyzed LTL and demonstrated that shorter LTL was independently associated with higher risks of hospitalization, critical care support, respiratory support, and death (*p* = 0.004), after adjustment for age, sex, and ethnicity [39]. However, older age (*p* < 0.001), male sex (*p* < 0.001), and non-White ethnicity (*p* < 0.001) were associated with even greater risk than TL alterations alone [39]. Vos et al. [40] reported a 10.12% decrease in TL among ICU patients compared with non-ICU patients [40]. Increased TL was associated with a lower risk of ICU admission (OR = 0.55), both before and after adjustment for age, BMI, education, ethnicity, Charlson comorbidity score, and smoking. Longer TL was also associated with greater resilience during COVID-19 disease progression, reflected by a 0.33-point reduction in severity after full covariate adjustment. Importantly, adjustment for blood cell counts demonstrated that leukocyte distribution shifts did not explain the association between longer TL and lower disease severity [40]. Froidure et al. [41] reported significantly shorter TL in hospitalized COVID-19 patients compared with a healthy reference population (*p* < 0.0001), with a markedly increased likelihood of hospitalization among individuals with TL below the 10th percentile [41]. Shorter TL was also associated with a higher risk of critical illness, defined as ICU admission or death without ICU admission (*p* = 0.02). Correlations were observed between TL and CRP (*p* = 0.03), neutrophil-to-lymphocyte ratio (*p* = 0.025), and lymphocyte count (*p* = 0.11), suggesting links between telomere attrition, inflammation, and immune dysfunction [41].

In summary, blood-based epigenetic clock measurements provide important insight into aging biology in critical illness, with growing evidence from the COVID-19 pandemic indicating that epigenetic age acceleration reflects host vulnerability and adverse disease trajectories. Mechanistic studies implicate epigenetic regulation of innate immune and inflammatory pathways in driving accelerated epigenetic aging during COVID-19 [42], and most clinical studies report a positive association between epigenetic age acceleration and severe outcomes [32,33,34,35,36].

By contrast, evidence linking TL to COVID-19 severity remains heterogeneous and inconclusive. While several studies found no overall TL differences between COVID-19 patients and controls [34,36,37], others reported that shorter TL was associated with higher risks of hospitalization, critical illness, or mortality, particularly in longitudinal or ICU-focused analyses [39,40,41]. Collectively, these data suggest that telomere shortening may capture pre-existing biological vulnerability in selected subgroups, whereas epigenetic clocks show more consistent associations with COVID-19 severity and outcomes.

### 4.5. Biological Aging Clocks Across Specific Pathologies in the ICU

#### 4.5.1. The Relationship Between Biological Aging and Psoriasis

In an observational cohort study [43] using the MIMIC-IV database, the authors evaluated the prognostic relevance of biological age acceleration in critically ill adults with psoriasis admitted to intensive care units in the United States. The cohort included 246 adults aged 18–90 years with a documented diagnosis of psoriasis and routine laboratory data available at ICU admission, reflecting a substantial comorbidity burden typical of ICU populations. The primary objective was to determine whether biological age acceleration at ICU admission was associated with short-term (28-day) mortality [43].

Biological age was calculated cross-sectionally at ICU admission using the PhenoAge and Klemera–Doubal Method Biological Age (KDM-age) clocks, both derived from standard laboratory biomarkers. PhenoAge acceleration was significantly associated with higher short-term mortality, with each unit increase corresponding to 13% higher odds of death (OR 1.13; 95% CI 1.09–1.18), whereas KDM-age showed no significant association. No additional ICU outcomes, including length of stay, duration of mechanical ventilation, or organ support requirements, were reported [43].

#### 4.5.2. Kidney-Specific Cell-Free DNA for Real-Time Monitoring of Sepsis-Induced AKI

In a prospective single-center observational study in China [44], the authors investigated whether kidney-specific cfDNA methylation markers could identify sepsis-induced acute kidney injury (SI-AKI) and distinguish it from sepsis without AKI. Using genome-wide methylation sequencing and cfDNA deconvolution, the study focused on kidney epithelial (kidney-Ep) and kidney endothelial (kidney-Endo) cfDNA fractions as early indicators of renal injury. The cohort comprised 98 individuals, including 82 healthy controls, seven patients with sepsis without AKI, and nine patients with SI-AKI according to KDIGO criteria. Plasma samples were collected at a single baseline point after enrollment [44].

Median kidney-Ep cfDNA concentrations were markedly higher in SI-AKI patients at 0.599 ng/µL (IQR 0.288–1.550) than in sepsis without AKI (0.026 ng/µL, IQR 0.017–0.076) or controls (0.014 ng/µL, IQR 0.010–0.019) (*p* < 0.001 for both comparisons) [44]. Kidney-Ep cfDNA correlated with total cfDNA levels (R = 0.58, *p* = 0.024) and tended to increase with more advanced SI-AKI stages. Kidney-Endo cfDNA concentrations were also higher in SI-AKI than in sepsis without AKI or controls, although the kidney-Endo fraction did not differ significantly between groups. However, kidney-Endo and kidney-Ep fractions were strongly correlated (R = 0.71, *p* = 0.0019). Diagnostic performance was high across cfDNA measures. Kidney-Ep cfDNA achieved an AUC of 0.9048, kidney-Endo cfDNA an AUC of 0.9365, combined kidney cfDNA (Ep + Endo) an AUC of 0.9206, and total cfDNA an AUC of 0.8889 (95% CI 0.7134–1.00). Notably, elevated kidney-Ep cfDNA was observed in some patients with sepsis without clinically defined AKI, suggesting potential detection of subclinical renal injury [44].

Clinically, these findings indicate that tissue-specific cfDNA methylation markers may identify SI-AKI earlier than functional criteria alone, with kidney-Ep cfDNA demonstrating high diagnostic accuracy. Biologically, the study shows that cfDNA methylome deconvolution captures cell-type–specific signals of organ injury beyond global cfDNA levels [44].

#### 4.5.3. Epigenomic Biomarkers in PBMCs for ECMO Cardiogenic Shock

In a prospective single-center study conducted in Taiwan [45], the authors investigated whether genome-wide DNA methylation patterns in PBMCs could predict short-term and in-hospital mortality among adults with cardiogenic shock requiring veno-arterial Extracorporeal Membrane Oxygenation (ECMO) support. DNA methylation profiling was performed at ECMO initiation, two hours after cannulation, and following ECMO removal to capture dynamic epigenetic responses to critical illness and mechanical circulatory support [45].

The cohort included 34 ECMO-treated patients (mean age 56.4 ± 14.8 years; 73.5% male). Patients with preexisting multiple organ failure, sepsis, severe brain injury, or inability to provide consent were excluded. Outcomes were assessed both at 7 days—classified as early success (survival > 7 days, n = 17) or early failure (death or multiple organ failure before day 7, n = 17)—and at hospital discharge, with survivors (n = 12) and non-survivors (n = 22) [45].

Genome-wide methylation analysis identified 314 CpGs associated with early failure at baseline and 428 CpGs at ECMO removal (FDR < 0.05). Methylation-based models demonstrated meaningful prognostic performance. The 10-CpG model predicted in-hospital mortality with an AUC of 0.78 (95% CI 0.62–0.93), while the 4-CpG model yielded an AUC of 0.72 (95% CI 0.55–0.88) [45].

In contrast, traditional ICU severity metrics, including APACHE II (median 32, *p* = 0.41), SOFA (median 12, *p* = 0.33), Survival After Veno-Arterial ECMO (SAVE) (median −11, *p* = 0.52), Multiple Organ Dysfunction Syndrome (MODS), and Logistic Organ Dysfunction Score (LODS), were not significantly associated with early failure or in-hospital mortality [45]. Early methylation changes also retained prognostic relevance, with 62 CpGs at the two-hour time point significantly associated with mortality (FDR < 0.05). Kaplan–Meier analysis using a methylation-based risk score showed a considerable reduction in survival among high-risk patients (*p* = 0.004). Mortality-associated CpG signatures were characterized by hypomethylation at enhancer regions and hypermethylation at immune-regulated promoters, suggesting heightened biological vulnerability under severe circulatory stress [45].

Overall, this study demonstrates that PBMC DNA methylation signatures are promising prognostic biomarkers in ECMO-supported cardiogenic shock and may outperform conventional ICU severity scores. These findings warrant validation in larger, multicenter cohorts and further refinement using cell-type–resolved epigenetic profiling [45].

#### 4.5.4. Accelerated Epigenetic Aging After Burn Injury

In a prospective observational study conducted in the United Kingdom [46], the authors investigated whether significant burn injury (>5% total body surface area [TBSA]) accelerates biological aging by analyzing DNA methylation–based epigenetic clocks in PBMCs. Blood samples were obtained at hospital admission and again six months later to assess both acute and medium-term effects of severe thermal trauma on biological age and the pace of aging [46].

The study enrolled 53 adult burn patients at admission (mean age approximately 45 years; mean TBSA of roughly 38%), of whom 34 were resampled at six months, along with 29 age-matched healthy controls [46]. Epigenetic aging was assessed using multiple clocks, including Horvath, Hannum, DNA methylation PhenoAge, Principal Component GrimAge (PCGrimAge), and the DunedinPACE pace-of-aging metric [46].

At admission, burn patients demonstrated marked acute epigenetic aging. DNA methylation PhenoAge exceeded chronological age by 7.20 years (*p* = 8.3 × 10^−5^), PCGrimAge by 9.23 years (*p* = 5.8 × 10^−11^), and DunedinPACE was increased by 31.65% (*p* = 2.1 × 10^−12^), corresponding to approximately 115 additional biological aging days per year. At six months, partial recovery was observed: PhenoAge acceleration normalized and no longer differed from controls, whereas PCGrimAge remained elevated by 4.18 years (*p* = 2.6 × 10^−6^) and DunedinPACE remained 11.36% higher than controls (*p* = 3.99 × 10^−5^), equivalent to approximately 41 excess aging days per year [46]. Horvath and Hannum clocks showed minimal or inconsistent changes across time points [46].

These findings suggest that major burn injury induces premature biological aging, potentially contributing to the increased long-term risks of cardiovascular disease, metabolic dysfunction, multimorbidity, and reduced survival observed in burn survivors. The sustained elevation of PCGrimAge and DunedinPACE indicates persistent epigenetic reprogramming, likely driven by chronic inflammation and immune dysregulation, reflecting a long-lasting increase in the pace of aging following critical injury [46].

#### 4.5.5. Epigenetic Biological Age in Aneurysmal Subarachnoid Hemorrhage

In a prospective observational study conducted at a tertiary stroke center in Spain [47], the authors investigated whether DNA methylation-based biological age at hospital admission is associated with major complications and outcomes following aneurysmal subarachnoid hemorrhage (aSAH). The study included 277 adult patients with aSAH, from whom peripheral blood samples were collected at admission for DNA methylation analysis. Biological age was estimated using multiple epigenetic clocks, including Hannum’s, Horvath’s, Levine’s, and both versions of Zhang’s clocks. Biological age acceleration (ageAcc) was defined as the residual of biological age regressed on chronological age. The primary objective was to assess whether epigenetic age acceleration predicted vasospasm, delayed cerebral ischemia (DCI), poor functional outcome (modified Rankin Scale [mRS] 3–5), or 12-month mortality, using multivariable regression models adjusted for relevant confounders [47]. The cohort had a mean age of 55.0 years, 66.8% were female, 51.6% developed vasospasm, 25.3% developed DCI, 35.7% had poor functional outcomes, and 20.6% died within 12 months [47].

The principal finding was a clock-specific association between biological age and outcomes. Older biological age estimated by Hannum’s clock was independently associated with higher 12-month mortality (OR 1.12 per 1-year increase in ageAcc; 95% CI 1.04–1.23; *p* = 0.006), whereas chronological age was not predictive [47]. In contrast, Horvath’s and Levine’s clocks demonstrated an inverse relationship with vasospasm risk, whereby younger biological age (negative ageAcc) was associated with a higher likelihood of vasospasm (Horvath: OR 0.89 per 1-year increase, 95% CI 0.81–0.97, *p* = 0.009; Levine: OR 0.91 per 1-year increase, 95% CI 0.84–0.99, *p* = 0.027) [47]. None of the epigenetic clocks showed a significant association with DCI after adjustment, and none meaningfully predicted poor functional outcome at 12 months (Hannum, *p* = 0.18; Horvath, *p* = 0.42; Levine, *p* = 0.35) [47].

Several additional findings informed the interpretation of these results. Both Zhang clocks exhibited high probe-missingness (>40%) on one of the methylation arrays used and were excluded from the primary analyses. Neither biological age nor chronological age was significantly associated with DCI, and chronological age was not related to vasospasm risk [47]. Overall, the observed associations followed distinct, clock-dependent patterns: higher biological age by Hannum’s clock predicted mortality, whereas lower biological age by Horvath’s and Levine’s clocks was paradoxically associated with vasospasm [47].

#### 4.5.6. 5-hmC Signatures in Septic Cardiomyopathy

In a prospective single-center cohort study conducted in China [48], the authors evaluated whether 5-hydroxymethylcytosine (5-hmC) patterns in extracellular vesicle-derived DNA (EV-DNA) could serve as early liquid-biopsy biomarkers for septic cardiomyopathy (SCM) in adults with sepsis [48]. The study included 39 patients: 13 with SCM, 18 with sepsis without myocardial dysfunction, and eight non-sepsis controls. Blood samples were collected at ICU admission, before clinical confirmation of cardiomyopathy, with the primary aim of assessing the early diagnostic value of EV-DNA 5-hmC signatures [48].

After EV-DNA analysis, differentially hydroxymethylated genes were identified, and pathway enrichment and clustering analyses were performed. A machine-learning classifier was developed to distinguish SCM from uncomplicated sepsis and was secondarily tested in two publicly available GEO datasets. The 5-hmC-based model demonstrated high diagnostic accuracy for SCM, achieving an AUC of 0.962, 92.3% sensitivity, and 88.9% specificity [48].

Secondary analyses showed that global hydroxymethylation levels increased progressively from controls to sepsis to SCM. Differential analyses identified extensive 5-hmC remodeling, with enrichment of pathways related to MAPK, JAK–STAT, NF-κB, PI3K–Akt, calcium signaling, apoptosis, and immune activation. Clustering analyses revealed coordinated epigenetic shifts across disease stages, characterized by upregulation of DNA-repair and stress-response pathways and downregulation of pathways involved in cytoskeletal organization and cellular motility [48].

Importantly, no associations were observed between 5-hmC patterns and clinical outcomes, including mortality, hemodynamic parameters, organ failure scores, or echocardiographic parameters. Overall, the study suggests that EV-derived 5-hmC signatures may enable early discrimination of SCM from uncomplicated sepsis at ICU admission and reflect the molecular progression of sepsis-induced myocardial dysfunction [48].

#### 4.5.7. Telomere/Telomerase System in Myocardial Infarction

In a cross-sectional clinical study from Serbia [49], the authors evaluated telomere biology as a marker of biological aging in acute coronary syndromes by measuring LTL and telomerase activity in patients with ST-elevation myocardial infarction (STEMI), stable angina, myocardial infarction with non-obstructive coronary arteries (MINOCA), coronary rupture, and healthy controls. Telomere biomarkers were assessed at hospital admission, immediately after primary percutaneous coronary intervention (PCI), and at six-month follow-up, together with oxidative stress indices, including the Protective score, Damage score, and oxidative stress index [49].

STEMI patients exhibited significantly shorter LTL compared with healthy controls (*p* < 0.001), accompanied by markedly higher telomerase activity (*p* < 0.001). Comparable telomere shortening was observed in MINOCA and coronary rupture groups (all *p* < 0.05 versus controls). Shorter LTL correlated with higher oxidative damage, while telomerase activity was positively associated with total cholesterol and LDL-C (*p* < 0.05) and inversely associated with HDL-C (*p* = 0.030) [49]. Following coronary reperfusion, STEMI patients showed a transient increase in TL, likely reflecting redistribution of younger leukocyte populations. In contrast, telomerase activity remained elevated at six months (*p* < 0.01), suggesting sustained activation of repair pathways in the context of persistent oxidative stress [49].

Analysis of thromboaspirate-derived cells revealed significantly longer telomeres (*p* < 0.01) and higher telomerase activity (*p* < 0.001) compared with peripheral blood leukocytes, linking telomere dynamics to the local thrombotic environment [49]. Multivariable regression identified telomerase activity (*p* = 0.036) and lipid parameters, including total cholesterol, HDL-C, and LDL-C (all *p* < 0.05), as independent predictors of LTL. Both LTL and telomerase activity effectively discriminated STEMI patients from healthy controls (*p* < 0.01), although AUC values were not reported [49].

#### 4.5.8. p16^INK4a as an Indicator of Biological Aging in Coronary Artery Bypass Grafting Patients

In a prospective pilot study from Johns Hopkins Hospital [50], the senescence marker Cyclin-Dependent Kinase Inhibitor 2A (p16^INK4a) was evaluated as a blood-based indicator of biological aging in older adults undergoing coronary artery bypass grafting, with particular interest in its relevance to postoperative recovery rather than as an isolated outcome predictor [50]. p16^INK4a expression was measured in peripheral blood T lymphocytes. Associations were explored with length of hospital and ICU stay, frailty, vascular stiffness, inflammatory status, cerebral oxygenation, and p16 protein expression in vascular tissues.

Forty-seven patients had complete p16^INK4a data. The cohort had a median age of 63.5 years and was predominantly male (74.5%). Median p16^INK4a mRNA expression in peripheral blood T lymphocytes was 4.71 (range 1.10–6.82) [50].

p16^INK4a expression was significantly associated with chronological age, increasing by 0.07 units per year (95% CI 0.02–0.17; *p* < 0.05), but showed no association with length of hospital stay (HR 1.10; 95% CI 0.87–1.40; *p* = 0.49), ICU stay (HR 0.98; 95% CI 0.74–1.28; *p* = 0.87), frailty, smoking, or exercise after age adjustment. Median IL-6 levels were 2.66 pg/mL (range 0.59–18.48), and IL-6 was positively associated with p16^INK4a expression in both univariate (β = 0.11; 95% CI 0.03–0.20; *p* < 0.05) and multivariable analyses (β = 0.09; 95% CI 0.01–0.18; *p* < 0.05), linking senescence marker expression to systemic inflammation [50].

Analysis of vascular tissue demonstrated marked heterogeneity in senescence across arterial beds. p16 protein expression was significantly higher in aortic tissue than in the left internal mammary artery, with a LIMA-to-aorta ratio of 0.49 (range 0.21–1.05; *p* < 0.0001), supporting the concept of tissue-specific aging. However, p16 levels in vascular tissue were not associated with clinical outcomes [50].

#### 4.5.9. GFAP as a Biomarker for Occult Intracranial Injury

In a prospective multicenter analysis from the TRACK-TBI study [51], researchers examined plasma glial fibrillary acidic protein (GFAP) as a biomarker of occult intracranial injury and outcome in patients with isolated traumatic subarachnoid hemorrhage (tSAH) after mild traumatic brain injury [51]. GFAP was selected for its established specificity for astroglial injury and prior evidence supporting its utility in detecting traumatic intracranial pathology not visible on computed tomography.

GFAP was measured in plasma within 24 h of injury in patients with isolated tSAH and correlated with structural findings on follow-up magnetic resonance imaging (MRI) and with short- and intermediate-term functional outcomes. Patients with CT-occult intracranial lesions identified on two-week MRI exhibited significantly higher GFAP concentrations than those without occult injury (median 630.6 pg/mL vs. 226.4 pg/mL; *p* = 0.049). This association was strongest for traumatic axonal injury, with GFAP levels markedly elevated in patients with axonal lesions compared with those without axonal injury (median 828.6 pg/mL vs. 226.7 pg/mL; *p* = 0.009). These findings support a specific relationship between astroglial injury, as reflected by GFAP release, and underlying axonal damage that is not detectable on initial CT imaging [51].

From a diagnostic perspective, GFAP demonstrated moderate discriminative performance for identifying CT-occult intracranial injury (AUC 0.653; 95% CI 0.503–0.802) and improved performance for detecting axonal injury specifically (AUC 0.729; 95% CI 0.491–0.968). Although the overall AUC values were below thresholds typically considered optimal for standalone diagnostics, the authors noted that the restricted sample size (n = 121) and focused inclusion criteria likely limited statistical power [51].

GFAP levels also aligned with functional recovery patterns. The presence of CT-occult injury associated with higher GFAP concentrations was linked to worse early outcomes, including higher rates of severe disability and lower rates of full recovery at two weeks and three months post-injury. By six months, outcome differences are attenuated, suggesting that GFAP may be most informative for identifying early vulnerability and incomplete recovery rather than long-term disability [51].

Overall, this study positions GFAP as a biologically plausible and clinically relevant biomarker that bridges the gap between radiographically “isolated” tSAH on CT and underlying microstructural brain injury detected on MRI. Elevated early GFAP levels appear to reflect astroglial and axonal injury burden, correlate with occult structural lesions, and identify patients at higher risk for delayed or incomplete recovery. These findings support the potential role of GFAP in refining triage strategies, guiding selective MRI use, and informing early risk stratification in patients with mild TBI and isolated tSAH, while underscoring the need for validation in larger cohorts [51].

### 4.6. Overview of Included Studies

Table 2 summarizes the key characteristics of the studies included in this scoping review, including population, biomarker domain, and primary outcome focus.

### 4.7. High-Yield Clinical Outcomes

To synthesize clinically actionable insights from heterogeneous biological aging studies in critical care, we summarized the most consistently reported, high-yield outcomes across major ICU phenotypes in Table 3. Rather than recapitulating methodological details, this table focuses on outcome-level associations between biological aging biomarkers and mortality, disease severity, organ dysfunction, and resource utilization. The findings are organized by clinical context to facilitate rapid interpretation and comparison across general ICU populations, sepsis, ARDS, COVID-19, and selected specific pathologies.

### 4.8. Limitations and Future Directions

#### 4.8.1. Current Limitations in the Application of Biological Clocks in the ICU

Despite growing evidence that biological aging metrics provide prognostic and mechanistic insight in critical illness, several consistent limitations constrain their interpretation and clinical translation. A major methodological limitation across ICU studies is the predominance of single-center, observational designs, which restrict external validity and increase susceptibility to selection bias. This is particularly evident in studies of epigenetic age acceleration and cfDNA in sepsis and ARDS, where inclusion frequently depended on the availability of specific laboratory parameters or biospecimens, potentially excluding the sickest or most unstable patients [11,12,13].

Another recurring limitation is reliance on single time-point sampling, typically at ICU admission. Biological aging measures—especially epigenetic clocks and proteomic stress signatures—are highly sensitive to acute inflammation and metabolic perturbation, complicating the distinction between chronic biological aging and transient stress-induced acceleration. This issue is highlighted in both COVID-19 and ARDS cohorts, where epigenetic age acceleration and proteomic alterations are partially normalized during recovery, while remaining persistently elevated in non-survivors [27,36]. Without longitudinal sampling, it remains unclear which signals represent reversible stress responses versus irreversible aging-related damage.

A further limitation concerns tissue specificity. Most studies derive biological age from peripheral blood, which may not accurately reflect aging processes in organs most relevant to ICU outcomes, such as lungs, kidneys, heart, or brain. This mismatch is evident in telomere and epigenetic studies, where blood-based measures failed to fully explain organ-specific injury or delayed recovery [15,37]. Similarly, proteomic and cfDNA analyses demonstrate that tissue-derived signals (e.g., kidney epithelial cfDNA in sepsis-induced AKI [42]) outperform global markers, yet such approaches remain underutilized.

Finally, heterogeneity of biological clocks remains a substantial barrier. Different epigenetic clocks capture distinct biological processes and may yield opposing associations within the same disease context, as demonstrated in aneurysmal subarachnoid hemorrhage, where younger biological age predicted vasospasm while older biological age predicted mortality depending on the clock used [47]. This underscores that clocks are not interchangeable and require mechanistic interpretation rather than uncritical aggregation.

Beyond biological and methodological constraints, the clinical application of biological aging clocks in the ICU faces substantial practical and ethical barriers. Current implementations often require complex laboratory workflows, specialized infrastructure, and non-trivial turnaround times, limiting their usability in time-critical decision-making. Cost and reimbursement considerations further restrict scalability. In parallel, the use of biologically derived risk estimates in critically ill or incapacitated patients raises ethical concerns related to informed consent, data governance, algorithmic bias, and the potential influence of such metrics on triage decisions, intensity-of-care choices, or perceptions of futility. These factors represent major obstacles to responsible and equitable clinical deployment.

#### 4.8.2. Future Directions and Actionable Paths Forward

Future research should prioritize multicenter, prospective ICU cohorts with standardized biospecimen collection, enabling external validation and reducing selection bias. Harmonized protocols for timing of sampling—at admission, during peak illness, and during recovery—are essential to resolve the temporal dynamics of biological aging and distinguish acute stress responses from persistent biological age acceleration. Longitudinal designs have already proven feasible and informative in burn injury, COVID-19, and ARDS cohorts and should be extended to broader ICU populations [27,36].

A second critical direction is the integration of tissue-specific aging signals. Approaches such as cfDNA methylation deconvolution, which can identify organ-specific injury, represent a ready-to-implement strategy that directly addresses blood–tissue discordance. Similarly, proteomic network analysis provides system-level insight into endothelial, immune, and metabolic dysfunction that cannot be captured by epigenetic clocks alone [27,28].

Third, network-based and multi-omic models should be favored over single-biomarker approaches. Recent ARDS studies demonstrate that integrated proteomic–metabolomic networks, particularly those centered on sphingolipid–MAPK and endothelial signaling pathways, outperform individual proteins or clinical severity scores in mortality prediction [27]. Importantly, these models are already operational, relying on serum-based assays and machine-learning frameworks that could be adapted for clinical research pipelines.

From a translational perspective, not all aging clock modalities are equally close to clinical implementation. Compared with epigenetic or multi-omic clocks requiring sequencing and complex bioinformatic pipelines, approaches based on routinely available laboratory data, targeted proteomic panels, or circulating cell-free DNA may represent more realistic near-term candidates for ICU integration, given their shorter turnaround times and lower technical barriers. Such modalities may serve as intermediate steps toward more comprehensive multi-omic implementations.

Biological clocks should initially be deployed as adjunctive risk-stratification tools rather than as standalone clinical decision-makers. Combining biological age metrics with established ICU scores (e.g., APACHE II, SOFA) may improve identification of high-risk patients, enrich clinical trial populations, and guide intensity of monitoring. This strategy is supported by studies showing that biological age retains prognostic value after adjustment for conventional severity scores [11,12].

Finally, future work should aim to establish reference frameworks for biological aging in critical illness that account for age, sex, ethnicity, comorbidities, and treatment exposures. The absence of standardized reference datasets currently limits interpretability and cross-study comparison. Constructing such reference atlases—analogous to population aging cohorts but tailored to ICU settings—would enable calibration of biological clocks and facilitate meta-analytic synthesis across studies.

#### 4.8.3. Limitations of the Present Scoping Review

Several limitations should be acknowledged when interpreting the findings of this scoping review. First, although the review followed a predefined PRISMA-ScR–based framework, no prospectively registered protocol was used, and no formal risk-of-bias assessment was performed, consistent with the aims and methodology of scoping reviews. Second, the available evidence base is highly heterogeneous and is dominated by small, single-center, observational studies, often with limited longitudinal sampling and variable adjustment for important confounders such as leukocyte composition, comorbidity burden, and pre-illness functional status.

Third, substantial methodological variability exists across biological aging measures, including differences in assay platforms, analytical pipelines, clock architectures, and timing of sample collection, which limits direct comparability across studies and precludes quantitative synthesis. Many clocks were developed and validated in non-ICU populations, and their performance, calibration, and biological interpretation under conditions of extreme acute stress remain incompletely understood.

In addition, this scoping review does not attempt to resolve the ethical, legal, and societal implications of deploying biological age-based tools in critical care, which will require dedicated normative and implementation-focused research alongside ongoing technical validation.

## 5. Conclusions

Personalization of critical care is increasingly recognized as essential not only for improving short-term survival but also for mitigating long-term morbidity and post-discharge functional decline among ICU survivors. Biological aging biomarkers provide a unifying framework for quantifying individual vulnerability, capturing latent physiological reserve, and bridging acute critical illness with downstream recovery trajectories.

Preclinical and translational studies presented in this review demonstrate that critical illness induces measurable alterations in molecular aging pathways, including epigenetic remodeling, telomere attrition, and dysregulated cell-free DNA dynamics. These changes are mechanistically linked to immune dysfunction, impaired tissue repair, endothelial injury, and persistent inflammation, supporting a causal role for biological aging acceleration in the pathophysiology of critical illness rather than a passive epiphenomenon.

Clinically, converging evidence across general ICU populations, sepsis, ARDS, COVID-19, and selected disease-specific cohorts indicates that biological aging metrics outperform chronological age and complement traditional severity scores in predicting mortality, organ failure, and resource utilization. Importantly, these biomarkers consistently identify high-risk phenotypes that are not apparent through conventional clinical assessment, underscoring their potential value for early risk stratification, prognostic enrichment, and individualized therapeutic decision-making.

Looking ahead, integrating biological aging biomarkers into critical care research and practice offers opportunities for precision phenotyping, dynamic monitoring of recovery versus maladaptive aging trajectories, and targeted interventional trials to preserve long-term physiological resilience. However, current limitations, including cohort heterogeneity, variable biomarker platforms, limited longitudinal sampling, and lack of standardized thresholds, preclude immediate routine bedside implementation of some of the biomarkers presented in this review.

In summary, biological aging represents a clinically meaningful construct that links molecular mechanisms to patient-centered outcomes in critical illness. Continued translational validation, harmonization of measurement approaches, and prospective interventional studies will be essential to establish biological age-guided strategies as a cornerstone of personalized critical care and improved post-discharge outcomes.

## Figures and Tables

**Table 1:** Biological aging clocks and molecular aging metrics identified in the reviewed literature.

Clock Category	Specific Clock/Marker
Epigenetic DNA methylation (DNAm) clocks	Horvath clock
	Hannum clock
	PhenoAge/PhenoAgeAccel
	GrimAge/PCGrimAge
	DunedinPACE
	Zhang-EN
	Zhang-BLUP
	KDM-age
Telomere-based and senescence clocks	Leukocyte telomere length (LTL)
	DNA methylation-based telomere length
	Telomerase activity
	p16^INK4a (mRNA)
	p16^INK4a (protein)
Cell-free DNA-derived clocks	Total plasma cfDNA
	Nuclear cfDNA
	Kidney epithelial cfDNA
	Kidney endothelial cfDNA
	EV-DNA 5-hmC signatures
Proteomic network clocks	8-protein ARDS mortality panel
Metabolomic aging markers	Sphingosine-1-phosphate (S1P)
	Lysophosphatidylcholine (LysoPC species)
Transcriptomic aging programs	9-gene ARDS mortality signature
	TP53-regulated transcriptional program
Phenotypic/laboratory-derived clocks	Molecular Age (MA score)
	Albumin-based molecular aging proxy
Neuro-injury-linked aging markers	GFAP

Abbreviations: DNAm—DNA methylation; PhenoAge—phenotypic age; PhenoAgeAccel—phenotypic age acceleration; GrimAge—DNA methylation-based mortality risk clock; PCGrimAge—principal component-based GrimAge; DunedinPACE—Dunedin Pace of Aging Computed from the Epigenome; Zhang-EN—Zhang elastic net model; Zhang-BLUP—Zhang best linear unbiased prediction model; KDM-age—Klemera–Doubal method biological age; LTL—leukocyte telomere length; mRNA—messenger RNA; cfDNA—cell-free DNA; EV—extracellular vesicle; 5-hmC—5-hydroxymethylcytosine; ARDS—acute respiratory distress syndrome; S1P—sphingosine-1-phosphate; LysoPC—lysophosphatidylcholine; TP53—tumor protein p53; MA—molecular age; GFAP—glial fibrillary acidic protein.

**Table 2:** Characteristics of included studies evaluating biological aging measures and related biomarkers in critical illness and acute disease states.

Study	Year	Cohort	Biomarker Domain	Main Outcome
Ho K.M. [11]	2024	Critically ill patients	Phenotypic biological age, frailty	Association with BMI and frailty
Anthony N.P. et al. [12]	2025	Critically ill patients	Phenotypic biological age, frailty	Hospital mortality
Ho K.M. et al. [13]	2023	Critically ill patients	Phenotypic biological age	Hospital mortality
Ho K.M. [14]	2023	Critically ill patients	Phenotypic biological age	ICU readmission
Zribi B. et al. [15]	2019	Critically ill patients	Telomere length	Telomere dynamics during critical illness
Van Dyck L. et al. [16]	2022	Critically ill patients	Epigenetic (DNA methylation)	Muscle DNA methylation alterations
Guang Y. et al. [17]	2022	Hospitalized patients	Phenotypic molecular age score	Survival probability
Lorente-Sorolla C. et al. [18]	2019	Sepsis patients	Epigenetic (monocyte DNA methylation)	Organ dysfunction, inflammation
Beltrán-García J. et al. [19]	2024	Severe sepsis patients	Epigenetic (leukocyte DNA methylation)	Immunosuppression, disease severity
Binnie A. et al. [20]	2020	Severe sepsis patients	Epigenetic (DNA methylation profiling)	Epigenetic profiling in sepsis
Sharma-Oates A. et al. [21]	2024	Critically ill patients	Epigenetic age	Clinical outcome
López-Cruz I. et al. [22]	2025	Septic and non-septic infection patients	Epigenetic (EWAS)	Sepsis biomarker identification
Rump K. et al. [23]	2019	Sepsis patients	Epigenetic (AQP5 promoter methylation)	Mortality
Liu S. et al. [24]	2020	Sepsis patients	Telomere length	Survival
Xu J. et al. [25]	2024	Sepsis patients	Telomere length	Causal association with sepsis
Cano-Gamez K. et al. [26]	2025	Sepsis patients	Cell-free DNA	Tissue origin and clearance of cfDNA
Lin M. et al. [27]	2024	ARDS patients	Proteomic/multi-omic	Mortality prediction
Liao S.Y. et al. [28]	2021	ARDS patients	Multi-omic	Mortality biomarkers
Zhang S. et al. [29]	2019	ARDS patients	Epigenetic/multi-omic	DNA methylation patterns
Bruse N. et al. [30]	2024	Critically ill COVID-19 patients	Clinical phenotyping	Survival associations
Cao X. et al. [31]	2022	COVID-19 patients	Epigenetic age	Disease severity
Andargie T.E. et al. [32]	2021	COVID-19 patients	Cell-free DNA	Tissue injury, mortality
Calzari L. et al. [33]	2023	COVID-19 patients	Epigenetic (EWAS)	Severe outcome
Corley M.J. et al. [34]	2021	COVID-19 patients	Epigenetic (DNA methylation)	Severe disease
Márquez-Salinas A. et al. [35]	2021	Severe COVID-19 patients	Accelerated aging metrics	Adverse outcomes
Bejaoui Y. et al. [36]	2023	COVID-19 ARDS patients	Epigenetic age	Mortality
Franzen J. et al. [37]	2021	COVID-19 patients	Epigenetic clocks	No acceleration detected
Wang Z. et al. [38]	2017	Viral infection (general)	Telomere biology	Telomeric response to infection
Wang Q. et al. [39]	2021	UK Biobank COVID-19 cohort	Telomere length	Adverse outcomes
Vos S. et al. [40]	2025	Hospitalized COVID-19 patients	Telomere length	Disease severity
Froidure A. et al. [41]	2020	COVID-19 patients	Telomere length	Severe disease risk
Salimi S. et al. [42]	2020	COVID-19 (conceptual)	Hallmarks of aging	Aging–COVID interactions
Lin Z. et al. [43]	2025	Psoriasis patients	Biological aging metrics	Disease association
You R. et al. [44]	2024	Sepsis-induced AKI patients	Cell-free DNA methylation	Kidney injury monitoring
Hsiao Y.-J. et al. [45]	2024	ECMO cardiogenic shock patients	Epigenetic biomarkers (PBMCs)	Prognosis
Sullivan J. et al. [46]	2025	Burn injury patients	Epigenetic age	Accelerated aging
Macias-Gómez A. et al. [47]	2024	Aneurysmal subarachnoid hemorrhage	Epigenetic biological age	Complications, outcomes
Zhen B. et al. [48]	2025	Septic cardiomyopathy patients	5-hmC epigenetic markers	Diagnosis
Vukašinović A. et al. [49]	2023	STEMI patients	Telomere–telomerase system	Oxidative stress association
Pustavoitau A. et al. [50]	2016	Post-CABG older adults	Senescence marker (p16INK4a)	Length of hospital stay
Yue J.K. et al. [51]	2024	Traumatic brain injury cohort	Clinical characterization	Phenotype of traumatic SAH

Abbreviations: ARDS, acute respiratory distress syndrome; AKI, acute kidney injury; cfDNA, cell-free DNA; EWAS, epigenome-wide association study; ICU, intensive care unit; PBMC, peripheral blood mononuclear cell.

**Table 3:** High-Yield Clinical Outcomes Associated With Biological Aging Biomarkers Across Critical Illness Phenotypes.

Discussion Section	High-Yield Clinical Outcome	References
General ICU outcomes	Biological age metrics capture frailty-related vulnerability that is not reflected in BMI or chronological age.	[11]
	PhenoAge acceleration independently predicts in-hospital mortality, outperforming chronological age and remaining significant after adjustment for APACHE II and comorbidities.	[12]
	Increasing biological–chronological age gap shows a dose–response relationship with mortality risk.	[13]
	Accelerated biological aging associates with unplanned ICU readmission during the same hospitalization.	[14]
Sepsis	AQP5 promoter CpG methylation (nt-937) is associated with higher 30-day mortality in sepsis (HR ≈ 3.3).	[23]
	Immune-gene DNA methylation markers improve prognostic discrimination for sepsis severity and outcome.	[22]
	Short LTL predicts worse 90-day and 1-year survival in septic patients.	[24]
	Short telomeres are associated with progression to severe ARDS among septic patients.	[24]
	Elevated plasma cfDNA correlates with organ failure severity and escalating organ support requirements.	[26]
ARDS	Multi-omic proteomic–metabolomic models provide robust mortality prediction in ARDS, outperforming single biomarkers.	[27]
	Angiopoietin-2 levels are associated with ARDS mortality, with moderate discriminative performance.	[28]
	A transcriptomic 9-gene signature predicts ARDS mortality with high accuracy.	[28]
	Short LTL predicts higher long-term mortality in ARDS.	[24]
COVID-19	Nuclear cfDNA levels discriminate patients requiring ICU-level care from non-critical COVID-19 cases.	[32]
	A 21-CpG epigenetic signature differentiates severe COVID-19 outcomes from controls.	[33]
	Positive PhenoAge acceleration associates with increased risk of adverse outcomes and mortality in COVID-19 patients.	[35]
	Epigenetic aging correlates with immune dysregulation and metabolic dysfunction in severe COVID-19.	[34]
Specific ICU pathologies	PhenoAge acceleration predicts 28-day mortality in critically ill patients with psoriasis.	[43]
	Kidney-specific cfDNA accurately identifies sepsis-induced acute kidney injury.	[44]
	PBMC DNA methylation signatures predict in-hospital mortality in ECMO-treated cardiogenic shock.	[45]
	Severe burns induce acute epigenetic age acceleration, with partial recovery over time.	[46]
	Epigenetic age acceleration shows clock-specific associations with mortality and vasospasm after aneurysmal subarachnoid hemorrhage.	[47]

Abbreviations: ICU—intensive care unit; PhenoAge—phenotypic age; APACHE II—Acute Physiology and Chronic Health Evaluation II; BMI—body mass index; AQP5—aquaporin 5; CpG—cytosine–phosphate–guanine dinucleotide; HR—hazard ratio; LTL—leukocyte telomere length; ARDS—acute respiratory distress syndrome; cfDNA—cell-free DNA; PBMC—peripheral blood mononuclear cells; ECMO—extracorporeal membrane oxygenation.

## Data Availability

All the data presented here can be reproduced based on Appendix A and is publicly available.

## Supplementary Materials

The following supporting information can be downloaded at: https://www.mdpi.com/article/10.3390/jpm16020092/s1, Supplemetory Material S1: Preferred Reporting Items for Systematic reviews and Meta-Analyses extension for Scoping Reviews (PRISMA-ScR) Checklist.

## Abbreviations

The following abbreviations are used in this manuscript:

5-hmC	5-Hydroxymethylcytosine
ANGPT2 (Ang-2)	Angiopoietin-2
AP-1	Activator Protein 1
APACHE II	Acute Physiology and Chronic Health Evaluation II
ARDS	Acute Respiratory Distress Syndrome
AUC	Area Under the Curve
BAX	BCL-2–Associated X Protein
BID	BH3-Interacting Domain Death Agonist
BMI	Body Mass Index
cfDNA	Cell-Free DNA
DCI	Delayed Cerebral Ischemia
DMP	Differentially Methylated Position
DMR	Differentially Methylated Region
ECMO	Extracorporeal Membrane Oxygenation
EV-DNA	Extracellular Vesicle DNA
FDR	False Discovery Rate
FLG2	Filaggrin-2
FBXO6	F-Box Protein 6
GFAP	Glial Fibrillary Acidic Protein
GWAS	Genome-Wide Association Study
HMGB1	High Mobility Group Box 1
INK4a (p16INK4a)	Cyclin-Dependent Kinase Inhibitor 2A
KDM-age	Klemera–Doubal Method Biological Age
LBP	Lipopolysaccharide-Binding Protein
LDHB	Lactate Dehydrogenase B
LMNA	Lamin A/C
LODS	Logistic Organ Dysfunction Score
LPC/LysoPC	Lysophosphatidylcholine
LTL	Leukocyte Telomere Length
MA	Molecular Age
MAP2K1	Mitogen-Activated Protein Kinase Kinase 1
MAPK	Mitogen-Activated Protein Kinase
MBL2	Mannose-Binding Lectin 2
MINOCA	Myocardial Infarction with Non-Obstructive Coronary Arteries
MODS	Multiple Organ Dysfunction Syndrome
MSN	Moesin
mRS	Modified Rankin Scale
NF-κB	Nuclear Factor Kappa-Light-Chain-Enhancer of Activated B Cells
PaO_2_/FiO_2_	Arterial Oxygen Partial Pressure to Fractional Inspired Oxygen Ratio
PBMC	Peripheral Blood Mononuclear Cell
PCDS	Pre-Chronic Disease Stage
PCGrimAge	Principal Component GrimAge
PBL-TL	Peripheral Blood Leukocyte Telomere Length
PCI	Percutaneous Coronary Intervention
PRISMA-ScR	Preferred Reporting Items for Systematic Reviews and Meta-Analyses—Extension for Scoping Reviews
qSOFA	Quick Sequential Organ Failure Assessment
RAGE	Receptor for Advanced Glycation End Products
SAVE	Survival After Veno-Arterial ECMO
SCM	Septic Cardiomyopathy
SEM	Standard Error of the Mean
SERPINA1	Serpin Family A Member 1
SI-AKI	Sepsis-Induced Acute Kidney Injury
S1P	Sphingosine-1-Phosphate
SOFA	Sequential Organ Failure Assessment
STAT	Signal Transducer and Activator of Transcription
TBSA	Total Body Surface Area
TL	Telomere Length
TP53	Tumor Protein p53
VEGF	Vascular Endothelial Growth Factor
VCAM1	Vascular Cell Adhesion Molecule-1

## Appendix A. Boolean Search Strategy Across PubMed, Scopus, Web of Science, and Embase

Pubmed:

(“biological age”[tiab] OR biologic age[tiab] OR epigenetic age[tiab] OR “epigenetic clock”[tiab] OR DNA methylation age[tiab] OR methylation clock[tiab] OR DNAm age[tiab] OR “age acceleration”[tiab] OR PhenoAge[tiab] OR GrimAge[tiab] OR DunedinPACE[tiab] OR deep aging clock[tiab] OR deep age predictor[tiab] OR deep biological age[tiab] OR AI aging clock[tiab] OR machine learning biological age[tiab] OR machine learning aging[tiab] OR deep learning aging[tiab] OR proteomic age[tiab] OR metabolomic age[tiab] OR transcriptomic age[tiab] OR multiomic clock[tiab] OR multiomic age[tiab] OR multi-omic clock[tiab] OR multi omic clock[tiab] OR “DNA Methylation”[Mesh]) AND (“intensive care”[tiab] OR “intensive care unit”[tiab] OR ICU[tiab] OR “critical care”[tiab] OR “critically ill”[tiab] OR “critical illness”[tiab] OR sepsis[tiab] OR septic shock[tiab] OR ARDS[tiab] OR organ failure[tiab] OR MODS[tiab] OR “Critical Illness”[Mesh] OR “Intensive Care Units”[Mesh] OR “Sepsis”[Mesh]) AND (“Adult”[Mesh] OR adult[tiab] OR adults[tiab] OR “aged”[Mesh]) AND humans[MeSH Terms] NOT (“Infant, Newborn”[Mesh] OR newborn[tiab] OR neonate[tiab] OR neonatal[tiab] OR perinatal[tiab]) NOT (Review[pt] OR Meta-Analysis[pt] OR Editorial[pt] OR Letter[pt] OR Comment[pt] OR Case Reports[pt] OR News[pt]) AND English[lang] AND (“2015/01/01”[dp]: “2025/11/04”[dp])

Scopus:

TITLE-ABS-KEY(“aging clock” OR “aging biomarkers” OR “biological clocks” OR “biological age” OR “biologic age” OR “biological aging” OR “deep longevity” OR “aging research” OR “deep learning aging” OR “biological age model” OR “AI aging” OR “aging predictor” OR “aging clocks” OR “epigenetic age” OR “epigenetic clock” OR “DNA methylation clock” OR “methylation clock” OR “age acceleration” OR “PhenoAge” OR “GrimAge” OR “DunedinPACE” OR “deep aging clock” OR “deep age predictor” OR “deep biological age” OR “machine learning biological age” OR “machine learning aging” OR “AI aging clock” OR “proteomic age” OR “metabolomic age” OR “transcriptomic age” OR “transcriptomic clock” OR “multi-omic clock” OR “multi omic clock” OR “multiomic clock” OR “multiomic age” OR “retinal images” OR “biological aging biomarker”) AND TITLE-ABS-KEY(“intensive care” OR “intensive care unit” OR “ICU” OR “critical care” OR “critically ill” OR “critical illness” or “sepsis” OR “septic shock” OR “organ failure” OR “multiple organ dysfunction” OR “multi organ failure” OR “organ dysfunction” OR “organ failure” OR “MODS” OR “acute respiratory distress syndrome” OR “ARDS”) AND PUBYEAR > 2014 AND PUBYEAR < 2026 AND (LIMIT-TO (DOCTYPE,“ar”)) AND (LIMIT-TO (LANGUAGE,“English”)) AND (EXCLUDE (EXACTKEYWORD,“Newborn”) OR EXCLUDE (EXACTKEYWORD,“Child”) OR EXCLUDE (EXACTKEYWORD,“Animal”))

Web of Science:

TS = ((“biological age” OR “epigenetic age” OR “epigenetic clock” OR “DNA methylation age” OR “methylation clock” OR “biological aging” OR “age acceleration” OR “deep aging clock” OR “AI aging clock” OR “machine learning aging” OR “deep learning aging” OR “proteomic age” OR “metabolomic age” OR “multi-omic clock” OR “multi omic clock”) AND (“intensive care” OR ICU OR “critical illness” OR “critically ill” OR “critical care” OR sepsis OR “organ failure” OR “organ dysfunction”)) AND TS = (“Adult” OR “Aged”) NOT TS = (“Infant” OR “Newborn” OR “Neonate”) AND LA = (English) AND DT = (Article) NOT DT = (Review OR “Book Chapter” OR “Meeting Abstract”) AND PY = (“2015” OR “2016” OR “2017” OR “2018” OR “2019” OR “2020” OR “2021” OR “2022” OR “2023” OR “2024” OR “2025”)

Embase:

(‘biological age’/exp OR ‘biological age’:ti,ab,kw OR ‘epigenetic age’/exp OR ‘epigenetic age’:ti,ab,kw OR ‘epigenetic clock’/exp OR ‘epigenetic clock’:ti,ab,kw OR ‘dna methylation age’/exp OR ‘dna methylation age’:ti,ab,kw OR ‘dna methylation clock’:ti,ab,kw OR ‘methylation clock’:ti,ab,kw OR ‘age acceleration’/exp OR ‘age acceleration’:ti,ab,kw OR ‘epigenetic age acceleration’:ti,ab,kw OR ‘biological age acceleration’:ti,ab,kw OR ‘phenotypic age’/exp OR ‘phenotypic age’:ti,ab,kw OR phenoage:ti,ab,kw OR phenoageaccel*:ti,ab,kw OR grimage:ti,ab,kw OR grim*age:ti,ab,kw OR grimageaccel*:ti,ab,kw OR dunedinpace:ti,ab,kw OR ‘dunedin pace’:ti,ab,kw OR dunedinpoam:ti,ab,kw OR poam:ti,ab,kw OR ‘pace of aging’:ti,ab,kw OR ‘deep aging clock’:ti,ab,kw OR ‘deep age predictor’:ti,ab,kw OR ‘machine learning biological age’:ti,ab,kw OR ‘ai aging clock’:ti,ab,kw OR ‘proteomic age’:ti,ab,kw OR ‘metabolomic age’:ti,ab,kw OR ‘transcriptomic age’:ti,ab,kw OR ‘multi-omic clock’:ti,ab,kw OR ‘multi omic clock’:ti,ab,kw OR ‘multiomic clock’:ti,ab,kw) AND (‘intensive care’/exp OR ‘intensive care’:ti,ab,kw OR ‘intensive care unit’/exp OR ‘intensive care unit’:ti,ab,kw OR icu:ti,ab,kw OR ‘critical care’/exp OR ‘critical care’:ti,ab,kw OR ‘critically ill’:ti,ab,kw OR ‘critical illness’/exp OR ‘critical illness’:ti,ab,kw OR ‘sepsis’/exp OR sepsis:ti,ab,kw OR ‘septic shock’:ti,ab,kw OR ‘organ failure’:ti,ab,kw OR ‘multiple organ dysfunction’:ti,ab,kw OR ‘ards’/exp OR ards:ti,ab,kw OR ‘acute respiratory distress syndrome’:ti,ab,kw) AND ‘article’/it AND ([adolescent]/lim OR [adult]/lim OR [young adult]/lim OR [middle aged]/lim OR [aged]/lim OR [very elderly]/lim) AND [humans]/lim AND [english]/lim AND [2015–2025]/py NOT (‘review’/it OR ‘systematic review’/it OR ‘meta analysis’/it OR ‘letter’/it OR ‘editorial’/it OR ‘conference abstract’/it OR ‘conference paper’/it OR ‘note’/it OR ‘comment’/it)
